# Identification of the sesquiterpene synthase AcTPS1 and high production of (–)-germacrene D in metabolically engineered *Saccharomyces cerevisiae*

**DOI:** 10.1186/s12934-022-01814-4

**Published:** 2022-05-18

**Authors:** Jiajia Liu, Chang Chen, Xiukun Wan, Ge Yao, Shaoheng Bao, Fuli Wang, Kang Wang, Tianyu Song, Penggang Han, Hui Jiang

**Affiliations:** State Key Laboratory of NBC Protection for Civilian, Beijing, 102205 People’s Republic of China

**Keywords:** Germacrene D, Sesquiterpene, Metabolic engineering, *Saccharomyces cerevisiae*, *Acremonium chrysogenum*

## Abstract

**Background:**

The sesquiterpene germacrene D is a highly promising product due to its wide variety of insecticidal activities and ability to serve as a precursor for many other sesquiterpenes. Biosynthesis of high value compounds through genome mining for synthases and metabolic engineering of microbial factories, especially *Saccharomyces cerevisiae*, has been proven to be an effective strategy. However, there have been no studies on the de novo synthesis of germacrene D from carbon sources in microbes. Hence, the construction of the *S. cerevisiae* cell factory to achieve high production of germacrene D is highly desirable.

**Results:**

We identified five putative sesquiterpene synthases (AcTPS1 to AcTPS5) from *Acremonium chrysogenum* and the major product of AcTPS1 characterized by in vivo, in vitro reaction and NMR detection was revealed to be (–)-germacrene D. After systematically comparing twenty-one germacrene D synthases, AcTPS1 was found to generate the highest amount of (–)-germacrene D and was integrated into the terpene precursor-enhancing yeast strain, achieving 376.2 mg/L of (–)-germacrene D. Iterative engineering was performed to improve the production of (–)-germacrene D, including increasing the copy numbers of *AcTPS1*, *tHMG1* and *ERG20*, and downregulating or knocking out other inhibitory factors (such as *erg9*, *rox1*, *dpp1*). Finally, the optimal strain LSc81 achieved 1.94 g/L (–)-germacrene D in shake-flask fermentation and 7.9 g/L (–)-germacrene D in a 5-L bioreactor, which is the highest reported (–)-germacrene D titer achieved to date.

**Conclusion:**

We successfully achieved high production of (–)-germacrene D in *S. cerevisiae* through terpene synthase mining and metabolic engineering, providing an impressive example of microbial overproduction of high-value compounds.

**Supplementary Information:**

The online version contains supplementary material available at 10.1186/s12934-022-01814-4.

## Background

**Fig. 1:**
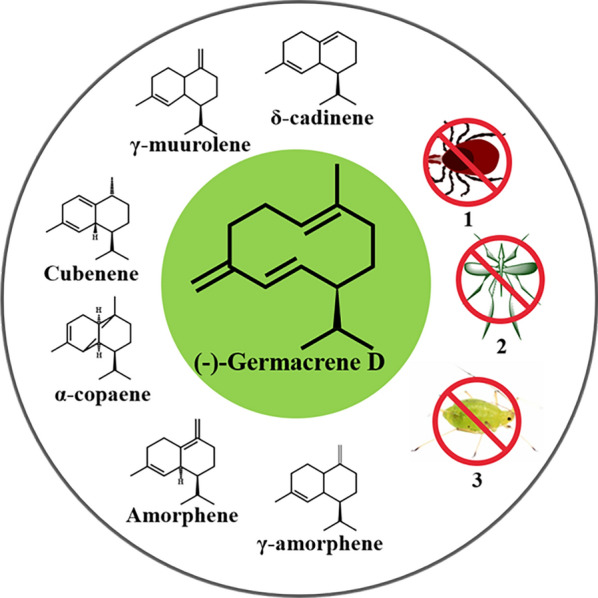
Sesquiterpenes obtained from germacrene D and bioactivity of (–) germacrene D. The known major sesquiterpenoids derived from germacrene D including γ-amorphene, amorphene, α-copaene, cubenene, γ-murolene and δ-cadinene. (–)-Germacrene D has repellent activity of anti-tick (1), anti-mosquito (2) and anti-aphid (3)

Sesquiterpenes are a large subgroup of terpenes with a wide range of applications; they have at least 121 different skeletons, including acyclic, monocyclic, bicyclic, tricyclic and tetracyclic skeletons, and have been identified in plants, bacteria, fungi and other organisms [1, 2]. Germacrene D is a monocyclic sesquiterpene. Since the first isolation of germacrene D from *Pseudotsuga japonica* in 1969, it has been widely found in many plants and in bryophytes [3, 4]. Like the most sesquiterpenes, germacrene D is a chiral compound. In higher plants the most common configuration was shown to be (–)-germacrene D [5]. Germacrene D is a high-value product due to its structural variability and insecticidal activity. As shown in Fig. 1, germacrene D is an important precursor of many other sesquiterpenes, such as cadinene and amorphene [5]. Germacrene D also has a variety of bioactivities including mosquito repellent, anti-aphid and anti-tick activities [6–8]. Like the most terpenoids obtaining germacrene D from natural sources with traditional extraction methods by using high amounts of energy and solvents may be unstable, resulting in shortages and price fluctuations [9]. Constructing the microbial cell factories to realize the high production of germacrene D is more promising in consideration of the short growth cycle and low costs. However, the de novo synthesis of (–)-germacrene D from carbon sources in microbes has not been realized.

Germacrene D was derived from the linear C15 precursor farnesyl diphosphate (FPP) through highly complex reactions catalyzed by germacrene D synthases [10]. Since the first germacrene D synthase was identified from *Lycopersicon esculentum* in 2000, 42 sesquiterpene synthases generating germacrene D have been identified to date (Additional file 1: Table S1). Most of the germacrene D synthase were cloned from plants and a small number have been found in microorganisms, including SCO6073 identified from bacteria *Streptomyces coelicolor A3(2)*, STC1 identified from fungi *Fusarium fujikuroi* and three sesquiterpene synthases Cop1, Cop2, Cop4 identified from mushroom *Coprinus cinereus* [11–13]. Actually, fungi have an excellent capacity for terpenoid product biosynthesis and therefore represent a promising resource for the discovery of new germacrene D synthases [14].

*Acremonium* fungi are a large group of ascomycetes which are widespread in soil, plants and marine environments, and have been proven to be a rich source of bioactive secondary metabolites [15]. A total of 86 terpenoids have been characterized in *Acremonium* fungi, thirty-one of which are sesquiterpenoids with different skeletons [15]. Four *Acremonium* fungal genome sequences have been released. The genome sequence of *Acremonium chrysogenum*, which is the most famous *Acremonium* fungi for producing the β-lactam antibiotic Cephalosporin C (CPC), was decoded and published in 2014 [16, 17]. Genome sequences of the other three *Acremonium* fungi were released very recently in 2021 and 2022. However, there have been very few reports on gene expression or functional characterization of sesquiterpene synthases from *Acremonium* fungi so far.

Notably, metabolic engineering makes it possible to express and identify fungal terpene synthases in *Saccharomyces cerevisiae* [18]. *S. cerevisiae* is a eukaryotic model organism used for genetic research and an attractive cell factory for biotechnological development [19]. In *S. cerevisiae* the sesquiterpene precursor FPP is biosynthesized through the mevalonate (MVA) pathway through the condensation of isopentenyl diphosphate (IPP) and dimethylallyl pyrophosphate (DMAPP) [10]. The introduction of sesquiterpene synthases to *S. cerevisiae* has made it possible to produce a variety of sesquiterpenes. The most common strategies for enhancing the production of high value terpenoids mainly focus on the MVA pathway in yeast [18]. For increasing the level of terpene precursors, strategies such as overexpressing the rate-limiting enzyme 3-hydroxyl-3-methylglutaryl-CoA reductase (tHMG1) or reconstructing the MVA pathway entirely have been used frequently [20]. In addition, downregulating the competing metabolic branches by deleting the squalene synthase gene (*ERG9*) or decreasing *ERG9* gene expression with inducible promoters is an effective strategy [21]. High production of sesquiterpenes in *S. cerevisiae* cell factories have been realized yet. The highest titer of linear sesquiterpene *β*-farnesene was achieved by Amyris, and the titer reached approximately 130 g/L [22]. In addition, the bicyclic sesquiterpene amorpha-4, 11-diene, the precursor of paclitaxel, was obtained at a titer of 40 g/L by metabolic engineering of *S. cerevisiae*. Recently, the sesquiterpene (–)-eremophilene was synthesized through expressing a synthase identified from *Ocimum sanctum* in *S. cerevisiae* and the titer achieved was 34.6 g/L, which was very close to the reported titer of amorpha-4,11-diene [23].

To achieve high production of (–)-germacrene D in microbes, we systematically analyzed the reported germacrene D synthases and sesquiterpene synthases from *A. chrysogenum*. A new (–)-germacrene D synthase, AcTPS1, was identified from *A. chrysogenum* through in vivo and in vitro analyses. *S. cerevisiae* was selected to construct cell factories to achieve high production of this molecule. We overproduced (–)-germacrene D by integrating three copies of AcTPS1, overexpressed the rate-limiting enzyme and repressed the competitive pathway in engineered yeast strains. Finally, the optimized strain LSc81 achieved a titer of 7.9 g/L (–)-germacrene D in fed-batch fermentation with 5-L bioreactors. This work highlights the great prospects of producing high value compounds through mining of the synthase and metabolically engineering of the microbial platform.

## Materials and methods

### Strains, plasmids, medium and chemicals

The strains and plasmids used in the study are listed in Additional file 1: Table S2. For yeast strain selection, SC medium (0.67% yeast nitrogen base, proper amino acid drop-out mix, 2% glucose) was used. For yeast strain growth, YPD medium (per liter, 10.0 g yeast extract, 20.0 g tryptone, 2% glucose) was used. YPDG medium (per liter, 10.0 g yeast extract, 20.0 g tryptone, 1% glucose, 1% galactose) was used for yeast shake-flask fermentation. Complete synthetic medium (CSM) (per liter, glucose 40 g, KH_2_PO_4_ 8 g, (NH_4_)_2_SO_4_ 15 g, MgSO_4_·7H_2_O 6.15 g, vitamin solution 12 mL, trace metals solution 10 mL; NH_3_·H_2_O used to adjust pH to 5.0) was used for fed-batch fermentation. CSM was supplemented with histidine (per liter, 0.1 g), uracil (per liter, 0.1 g), tryptophan (per liter, 0.1 g) and leucine (per liter, 0.5 g) for auxotrophic recombinant strain growth. Vitamin solution (per liter, biotin 0.05 g, calcium pantothenate 1 g, nicotinic acid 1 g, myo-inositol 25 g, thiamine · HCl 1 g, pyridoxal · HCl 1 g, and p-aminobenzoic acid 0.2 g) and trace metal solution (per liter, EDTA 15 g, ZnSO_4_·7H_2_O 5.75 g, MnCl_2_·4H_2_O 0.32 g, CuSO_4_ 0.5 g, CoCl_2_·6H_2_O 0.47 g, Na_2_MoO_4_·2H_2_O 0.48 g, CaCl_2_·2H_2_O 2.9 g, FeSO_4_·7H_2_O 2.8 g) were individually tailored. Feeding solution I (per liter, 500 g/L glucose, 9 g/L KH_2_PO_4_, 5.12 g/L MgSO_4_, 3.5 g/L K_2_SO_4_, 0.28 g/L, Na_2_SO_4_) was used for fed-batch fermentation. DNA polymerase was purchased from Takara (Beijing, China). Restriction endonucleases were purchased from Thermo Fisher Scientific (Waltham, MA). T4 ligase, Q5 DNA Polymerase and Gibson assembly enzyme was purchased from New England BioLabs (Ipswich, MA). The primers were purchased from GENEWIZ (Tianjin, China). (–)-Germacrene D (Toronto Research Chemicals, #G367765, CAS: 23986–74-5) was used as standard.

### Bioinformatic analysis of *A. chrysogenum* sesquiterpene synthases and cloning of the sesquiterpene gene coding sequences from *A. chrysogenum*

Using the BLASTP program, the whole genomic protein sequence of *A. chrysogenum* ATCC 11,550 was searched against the nonredundant protein database of the National Center for Biotechnology Information (NCBI). The amino acid sequences of the five sesquiterpene synthases (AcTPS1, AcTPS2, AcTPS3, AcTPS4 and AcTPS5) from *A. chrysogenum* and the germacrene D synthases were aligned by ClustalW. The conserved motifs of *A. chrysogenum* sesquiterpene synthases were subsequently analyzed by ESPript 3 [24]. The bootstrapped maximum-likelihood phylogenetic trees were generated using the MEGA 11 program based on the Jones-Taylor-Thornton matrix-based model [25].

To clone *Actps1*, *Actps2*, *Actps3*, *Actps4* and *Actps5*, *A. chrysogenum* WT was grown in TSA liquid medium at 28 °C. After 48 h of incubation, the mycelia were harvested and dried with filter paper. Then the mycelia were ground in liquid nitrogen. Using the DNA Quick Plant System (TianGen, China) and Trizol Reagent (Invitrogen, USA), the genomic DNA and the total RNA were isolated, respectively. With reverse transcription of total RNA using the PrimeScript™ RT reagent kit (TaKaRa, Japan), the cDNA of *A. chrysogenum* was obtained. The DNA and cDNA of *Actps1*, *Actps2*, *Actps3*, *Actps4* and *Actps5* were amplified with the RT-Actps1-F/R, RT-Actps2-F/R, RT-Actps3-F/R, RT-Actps4-F/R and RT-Actps5-F/R primers respectively. Then, the DNA fragments were verified by sequencing (GENEWIZ, China).

### Construction of the plasmids and yeast strains

The primers used in this study are listed in Additional file 1: Table S3. The plasmids pLJJ1, pLJJ2, pLJJ3, pLJJ4 and pLJJ5 were constructed for self-replicating the expression of *Actps1-5*. Q5 DNA Polymerase was used for amplifying DNA fragments. The codon-optimized gene sequences of *Actps1-5* were synthesized by BGI (Beijing, China).

For the construction of pLJJ1, the LB of *ROX1*, terminator of *CYC1*, promoter of *GAL1* and RB of *ROX1* were amplified with the primers pLJJ1-1-F/R, pLJJ1-2-F/R, pLJJ1-4-F/R and pLJJ1-5-F/R using the *CEN.PK2-1D* genomic DNA as templates, respectively. *Actps1* was amplified with the primers pLJJ1-3-F/R using the synthesized DNA sequence as a template. The plasmid backbone was amplified with the primers pLJJ1-6-F/R using pRS426 as a template. Finally, these six fragments were assembled by Gibson Assembly to generate pLJJ1. For the construction of pLJJ2 to pLJJ5, the LB of *ROX1*, terminator of *CYC1*, promoter of *GAL1*, RB of *ROX1* and plasmid backbone were the same as those used for pLJJ1. *Actps2* to *Actps5* were amplified with the primers pLJJ2-3-F, pLJJ3-3-F, pLJJ4-3-F, and pLJJ5-3-F, from the synthesized DNA sequence, respectively. Finally, the above amplified fragments were assembled to generate pLJJ2 to pLJJ5.

The *Actps1* DNA fragment was amplified with pLJJ6-F/R and inserted into the *Sac*I site of pET-45b to generate the plasmid pLJJ6. For the construction of pLJJ7, the primers pLJJ1-1-F/R, pLJJ1-2-F/R, pLJJ1-4-F/R, pLJJ1-5-F/R and pLJJ7-F/R were used to amplify the LB of *ROX1*, terminator of *CYC1*, promoter of *GAL1*, RB of *ROX1* and DNA fragment of *SSLH2*. The above fragments were assembled by overlap PCR and cloned into pEASY-Blunt to generate pLJJ7. The plasmids from pLJJ8 to pLJJ26 were constructed similarly as pLJJ7 except exchanging the primers pLJJ7-F/R with pLJJ8-F/R to pLJJ26-F/R to amplify the related cDNA fragments of germacrene D synthases. The fragments constructed for pLJJ1 were assembled with overlap PCR and cloned into pEASY-Blunt to generate pLJJ27.

For construction of the plasmid pLJJ28 the sgRNA of *ROX1* was amplified with the primers pLJJ28-1-F/R using sgRNA as a template. The truncated *URA3* was amplified from plasmid KlURA3 with primers pLJJ28-2-F/R. Finally the above fragments were cloned into the *Bsa*I site of pCAS by Golden Gate cloning. The sgRNAs of *exg1* or *dpp1* was amplified with the primers pLJJ30-1-F/R or pLJJ30-3-F/R using plasmid SgRNA as a template. The truncated *URA3* was amplified from KlURA3 with the primers pLJJ30-2-F/R. The above fragments were cloned into the *Bsa*I site of pCAS by Golden Gate cloning. The sgRNA of *EGR9* was amplified with the primers pLJJ31-1-F/R using SgRNA as a template. The truncated *URA3* was amplified from KlURA3 with the primers pLJJ31-2-F/R. Finally the above fragments were cloned into the *Bsa*I site of pCAS by Golden Gate cloning to generate the plasmid pLJJ31.

For construction of the plasmid pLJJ29, the LB of *ROX1* and terminator of *CYC1* were amplified from *CEN.PK2-1D* genomic DNA with the primers pLJJ1-1-F/R and pLJJ1-2-F/R, respectively. DNA fragment of *Actps1* and the promoter of *GAL1*, *GAL10* were amplified with the primers pLJJ1-3-F/R, pLJJ1-4-F/pLJJ29-4-R from synthesized DNA sequence of *Actps1* and *CEN.PK2-1D* genome DNA, respectively. The terminator of *GAL10* and promoter of *GAL7* was amplified with the primers pLJJ29-6-F/R using *CEN.PK2-1D* genomic DNA as a template. *ERG20*, *tHMG1*, the terminator of *ADH1* and the RB of *ROX1* were amplified with the primers pLJJ29-5-F/R, pLJJ29-7-F/R, pLJJ29-8-F/R, pLJJ29-9-F/R respectively. The plasmid backbone was amplified with the primers pLJJ1-6-F/R using pRS426 as a template. Finally, these fragments were assembled by Gibson assembly to generate pLJJ29.

For construction of the plasmid pLJJ32, the LB of *exg1* and terminator of *CYC1* were amplified with the primers pLJJ32-1-F/R and pLJJ32-2-F/R from *CEN.PK2-1D* genomic DNA. *tHMG1*, the promoter of *GAL1*, *GAL10*, and the DNA sequence of *Actps1* were amplified with the primers pLJJ32-3-F/R, pLJJ32-4-F/R, pLJJ32-5-F/R. The terminator of tPGK1, RB of *exg1* were amplified with primers pLJJ32-6-F/R, pLJJ32-7-F/R from *CEN.PK2-1D* genomic DNA. The plasmid backbone was amplified with the primer pLJJ32-8-F/R using pRS426 as a template. Finally, these above fragments were assembled by Gibson assembly to generate pLJJ32.

For construction of the plasmid pLJJ33, the LB of *dpp1* and terminator of *CYC1* were amplified with the primers pLJJ133-1-F/R and LJJ133-2-F/LJJ132-2-R from *CEN.PK2-1D* genomic DNA. *tHMG1*, the promoter of *GAL1* and *GAL10*, and the DNA of *Actps1* were amplified with the primers pLJJ32-3-F/R and pLJJ32-4-F/R, pLJJ32-5-F/R. The terminator of tPGK1, the RB of *dpp1* were amplified with primers pLJJ32-6-F/ pLJJ133-7-R and pLJJ133-8-F/R from *CEN.PK2-1D* genomic DNA. The plasmid backbone was amplified with the primers pLJJ133-9-F/R using pRS426 as a template. Finally, these fragments were assembled by Gibson assembly to generate pLJJ33.

For construction of the plasmid pLJJ34, the LB of *ERG9*, RB of *ERG9*, promoter of *HXT1* were amplified with primers pLJJ34-1-F/R, pLJJ34-3-F/R and pLJJ34-2-F/R from *CEN.PK2-1D* genomic DNA. The fragments were assembled with overlap PCR and cloned into pEASY-Blunt to generate pLJJ34.

JCR27 was used as initial strain. LSc1 to LSc5 were obtained by transforming plasmids pLJJ1-5 into JCR27 by the PEG/LiCl method, and strains were selected by uracil defective SC plates and confirmed by colony-PCR. The correct recombinant strains were saved as LSc1 to LSc5.

SC1 to SC21 were obtained by integrating the expression cassette into the *ROX1* site of JCR27. The plasmids pLJJ7-pLJJ27 were digested by *Pme*I. Fragment with an expression cassette for germacrene D synthases were purified and transformed into the JCR27 strain by PEG/LiCl method together with the plasmid pLJJ28. The recombinant strains were selected by uracil defective SC plates and confirmed by colony PCR. The correct strains were screened with a 5-FOA SC plate for plasmid loss. The final correct strains were saved as SC1 to SC21.

For construction of LSc53, the plasmid pLJJ29 was digested by *Pme*I. Fragment with *Actps1* expression cassette were purified and transformed into JCR27 together with the plasmid pLJJ28. Strains were selected by uracil defective SC plates and verified by colony PCR; The correct strains were screened with a 5-FOA SC plate for plasmid loss. The finally correct strains were saved as LSc53.

For the construction of LSc54, another two copies of the *Actps1* expression cassette were obtained by digesting the plasmids pLJJ32 and pLJJ33 with *Pme*I. Fragments with the *Actps1* expression cassette were purified and transformed into LSc53 together with the plasmid pLJJ30. Strains were selected by uracil defective SC plates and verified by colony PCR. The correct strains were screened with a 5-FOA SC plate for plasmid loss. The finally correct strains were saved as LSc54.

LSc81 was obtained by inserting the fragment expressing *ERG9* under the control of P_HXT1_. The plasmid pLJJ34 was digested with *Pme*I. The fragment with *ERG9* expression cassette was purified and transformed into LSc54 together with the plasmid pLJJ31. Strains selected by uracil defective SC plates and verified by colony PCR; The correct strains were screened with a 5-FOA SC plate for plasmid loss; The finally correct strains were saved as LSc81.

### Functional characterization in *Escherichia coli*

The plasmid pLJJ6 was transformed into *E. coli Rosetta* to generate strain LEc1. LEc1 was cultured at 37 ℃; 0.5 mM isopropyl-β-d-thiogalactoside (IPTG) was added when the OD_600_ reached approximately 0.6 to induce recombinant protein expression, and the cells were cultured at 16 ℃ overnight. Then the transgenic cells were collected and lysed in Buffer A (20 mM Tris–HCl, 500 mM sodium chloride with 5 mM imidazole; pH 7.9) by ultrasonication. The cell debris was removed by centrifugation. The supernatant protein was purified by a Ni–NTA affinity chromatography column and assayed by SDS-PAGE.

An in vitro enzyme assay was performed in a total volume of 500 μL reaction buffer (50 mM Tris–HCl) containing 2 mM Mg^2+^, 200 μL of cell crude extracts and 50 μM FPP at 30 ℃ for 2 h. The reaction mixture was overlaid with 500 μL of hexane, and then hexane was collected for GC–MS analysis. The negative control with crude extracts of empty vector cells replacing the recombinant protein cell crude extracts was also subjected to GC–MS analysis.

### Shake-flask fermentation

For shake-flask fermentation, the recombinant strains were revitalized on YPD plates. Then monoclonal was inoculated into 5 mL YPD medium at 30 ℃ for 16–18 h. 0.5 mL cultures were subsequently added into 50 mL of YPDG medium in a 250 mL flask and cultured at 30 ℃ for 3 days of fermentation subsequently. decane (10%) was added to the cultures as an organic extractant for in situ extraction when the OD_600_ reached approximately 0.8–1.

### Fed-batch fermentation

The medium used for fed-batch fermentation was derived as described previously [23]. Fed-batch fermentation was performed in a 5-L bioreactor (Shanghai Baoxing Biological Equipment Engineering Co., Ltd., China). The engineered strain was cultured in 5 mL YPD medium overnight at 30 ℃ and 220 rpm overnight. Then 1% of the culture was transferred to a 500 mL flask with 200 mL YPD medium and cultured at 30 ℃, 220 rpm for 16 h-18 h as the seed culture. Ten percent of the seed culture was added to 2000 mL medium in a 5-L fermenter for fed-batch fermentation at 30 ℃. The pH, dissolved oxygen content, and temperature were measured with online detectors. The pH was maintained at 5.0 by automatic addition of NH_3_·H_2_O. The dissolved oxygen content was controlled at approximately 10–30% by adjusting the stirring rate and airflow rate. The temperature was automatically controlled at 30 ℃. A two-stage strategy was used for fed-batch fermentation. At the first stage, feeding solution I was added to ensure rapid cell growth while the residual glucose was below 1 g/L and to control the glucose concentration around 1 g/L. When the growth curve flattened, the fermentation switched to the second stage by adding 500 mL isopropyl myristate and galactose (w/v 1%). Meanwhile 400 mL YPD with additional histidine (per liter, 0.1 g), uracil (per liter, 0.1 g), tryptophan (per liter, 0.1 g) and leucine (per liter, 0.5 g) were added to the fed-batch cultures to ensure the further growth of auxotrophic strains. Ethanol was added when the ethanol concentration was below 5 g/L and used as feeding solution II to control the ethanol residual concentration at an appropriate level. The ethanol and glucose were determined by Bioanalyzer (SBA-40C, Shandong Academy of Sciences, China) [23]. The fermentation was deemed to be complete when the target product titer stopped increasing.

### Extraction and analysis of the AcTPS1 product

To determine the chemical structure of the product of AcTPS1, the decane fraction overlaid on the shake-flask fermentation culture was collected and initially purified by reduced pressure distillation. Then the fractions were confirmed by GC–MS. The refined compound was obtained using an Ultimate 3000 HPLC equipment with an RP C18 column and analyzed by NMR. CDCl_3_ was used for NMR measurements. The optical rotation was analyzed by Rudolph Research Analytical Autopol IV Automatic Polarimeter in CDCl_3_ solution.

For quantification of the production of germacrene D, GC–MS (Agilent Technologies 7890A gas chromatograph equipped with 5975C inert XL MSD) assessment was performed. The oven temperature was initially held at 50 °C for 1 min and was then increased at a rate of 50 °C/min to 100 °C, where it was held for 1 min. Then, the oven temperature was increased at a rate of 20 °C/min to 280 °C, where it was held for 2 min. Nitrogen was used as the carrier gas with an inlet pressure of 39 psi. The products were analyzed in total ion monitoring mode.

## Results and discussion

### Bioinformatic analysis of *A. chrysogenum* sesquiterpene synthases and germacrene D synthase

To identify the germacrene D synthase homolog in the *Acremonium* fungi, a BLAST search of the *Acremonium* genomic database (taxid: 159075) was performed using the sequence of germacrene D synthase STC1 (GenBank Accession No. XM_023573743.1) from *Fusarium fujikuroi*. Based on the bioinformatic analysis, we found one homolog from *A. chrysogenum* ATCC11550, and it was designated AcTPS1 (GenBank Accession No. KFH47455.1, predicted to be a presilphiperfolan-8-beta-ol synthase-like protein). This suggested that AcTPS1 might be a homologue of STC1. Since fungal genome sequences present duplicate putative terpene synthases [13], a comprehensive analysis of other sesquiterpene synthases in *A. chrysogenum* ATCC11550 was conducted. Using characterized microbial sesquiterpene synthases for a BLAST analysis of the *A. chrysogenum* genomic database (taxid: 857340) resulted in the identification of AcTPS1 and four additional synthases, which were designated AcTPS2 (GenBank Accession No. KFH45267.1, predicted to be a microbial Terpene synthase-like protein), AcTPS3 (GenBank Accession No. KFH47841.1, predicted to be a presilphiperfolan-8-beta-ol synthase-like protein), AcTPS4 (GenBank Accession No. KFH40651.1, predicted to be a hypothetical protein ACRE_086550) and AcTPS5 (GenBank Accession No. KFH42720.1, predicted to be a trichodiene synthase-like protein).

As shown in Additional file 1: Fig. S1, all five synthases have the conserved terpene synthase domain NSE/DTE and the amino acid (aa) sequence lengths are 373 aa, 498 aa, 383 aa, 302 aa and 390 aa, respectively. It is worth noting that the DDXXD/E domain is absent in AcTPS4 and AcTPS5, but in AcTPS1, there are two DDXXD/E domains as _103_DDVTD_107_ and _184_DDVED_188_, respectively (Additional file 1: Fig. S1). Among the five putative sesquiterpene synthases AcTPS2, AcTPS4 and AcTPS5 shows low identity with STC1, and AcTPS1 and AcTPS3 shows 45% and 25% identity to STC1, respectively (Additional file 1: Fig. S2).

**Fig. 2:**
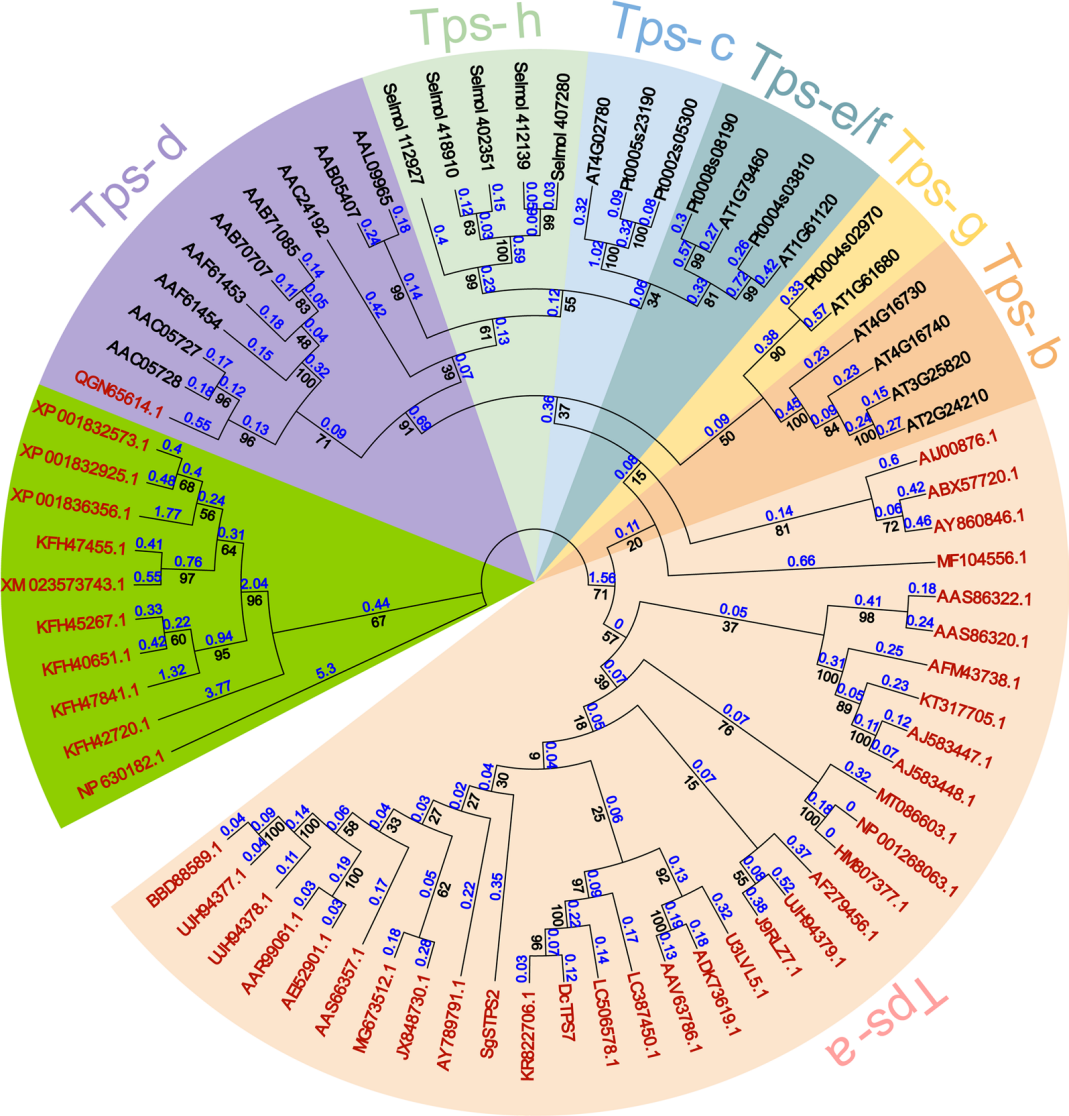
Phylogenetic analysis of *A. chrysogenum* sesquiterpene synthases and germacrene D synthases. The TPS-a, TPS-b, TPS-c, TPS-d, TPS-e/f, TPS-g, TPS-h clades and the microbial branching are shown in different colors. *A. chrysogenum* sesquiterpene synthases and germacrene D synthases are shown in red font. Terpene synthases of *Arabidopsis thaliana*, *Selaginella moellendorffii Abies grandis*, *Populus trichocarpa* and *Ginkgo biloba* were used for phylogenetic analysis and are shown in Additional file 1: Table. S4. The branch lengths and bootstrap values are presented at the nodes in blue and black, respectively

To gain insight into the evolutionary relationship of *A. chrysogenum* putative sesquiterpene synthases and germacrene D synthases, a phylogenetic analysis was performed. The sequences of the above five sesquiterpene synthases, all the reported germacrene D synthases and some other terpene synthase sequences were retrieved from the public database as reference (Additional file 1: Tables S1, S2). The results of phylogenetic analysis showed that the *A. chrysogenum* sesquiterpene synthases and germacrene D synthases were clustered into three subclades, TPS-a, TPS-d, and the microbial branch (Fig. 2). Almost all the germacrene D synthases identified from plants were belonged to the TPS-a subclade. Sesquiterpene synthases identified from *Solidago canadensis* (GenBank Accession No. AJ583448.1 and AJ583447.1), *Pogostemon cablin* (GenBank Accession No. AAS86320.1), *Matricaria chamomilla* (GenBank Accession No. AFM43738.1), and *Xanthium strumarium* (GenBank Accession No. KT317705.1) were clustered together in the TPS-a subclade (Fig. 2). These synthases produced germacrene D as mostly a single product (Additional file 1: Table. S1). The synthase TcTPS8 (GenBank Accession No. QGN65614.1) cloned from the gymnosperm *Taiwania cryptomerioides* was belonged to TPS-d as previously reported [26]. The synthase SCO6073 (GenBank Accession No. NP_630182.1) identified from *Streptomyces coelicolor* A3(2) was separated from the others because *Streptomyces* is a prokaryotic organism. Synthases identified from fungi were clustered together. AcTPS1 was close to germacrene D synthase STC1 and evolved together with synthases identified from *C. cinereus* (Fig. 2), suggesting that they may catalyze the same or related cyclization reaction.

### Cloning and characterization of sesquiterpene synthases of *Acremonium chrysogenum*

To confirm the gene sequences of the putative sesquiterpene synthases from *A. chrysogenum*, gene *Actps1* to *Actps5* were amplified from genomic DNA based on the gene predictions. As a result, all five genes were cloned successfully and intact (from the initiation codon to the termination codon) (Additional file 1: Fig. S3). Considering that the *A. chrysogenum* has an intron-rich genome and the inaccuracy of computational gene cDNA prediction [17], coding sequences of the five genes were amplified from cDNA. According to the results, only *Actps1* and *Actps5* showed bands by PCR using cDNA as a template (Additional file 1: Fig. S3), suggesting that the other genes were not transcribed or were transcribed weakly under laboratory cultivation conditions in *A. chrysogenum*. Therefore, we synthesized codon-optimized sequences of *Actps1* and *Actps5* based on the cDNA sequence. The expression sequences of *Actps2, Actps3* and *Actps4* were synthesized based on the predicted CDS.

The terpene precursor-producing yeast strain JCR27 was used to characterize the predicted sesquiterpene synthases AcTPS1-5. JCR27 contained the additional copies of MVA pathway related enzymes, including ERG10 (acetyl‐CoA acetyl transferase), ERG13 (3‐hydroxy‐3‐methylglutaryl CoA synthase), tHMG1, ERG12 (mevalonate kinase), ERG8 (phosphomevalonate kinase), MVD1 (mevalonate diphosphate decarboxylase), and IDI (isopentenyl diphosphate isomerase) [27]. *Actps1* to *Actps5* were expressed under the control of *GAL1* promoter and their expression was induced for expression with galactose. The plasmids pLJJ1-5 containing the expression cassette of *Actps1-5* were transformed into JCR27 to obtain expressing strains LSc1 to LSc5, respectively. The strains were fermented in YPDGH medium, and products were detected by GC–MS.

**Fig. 3:**
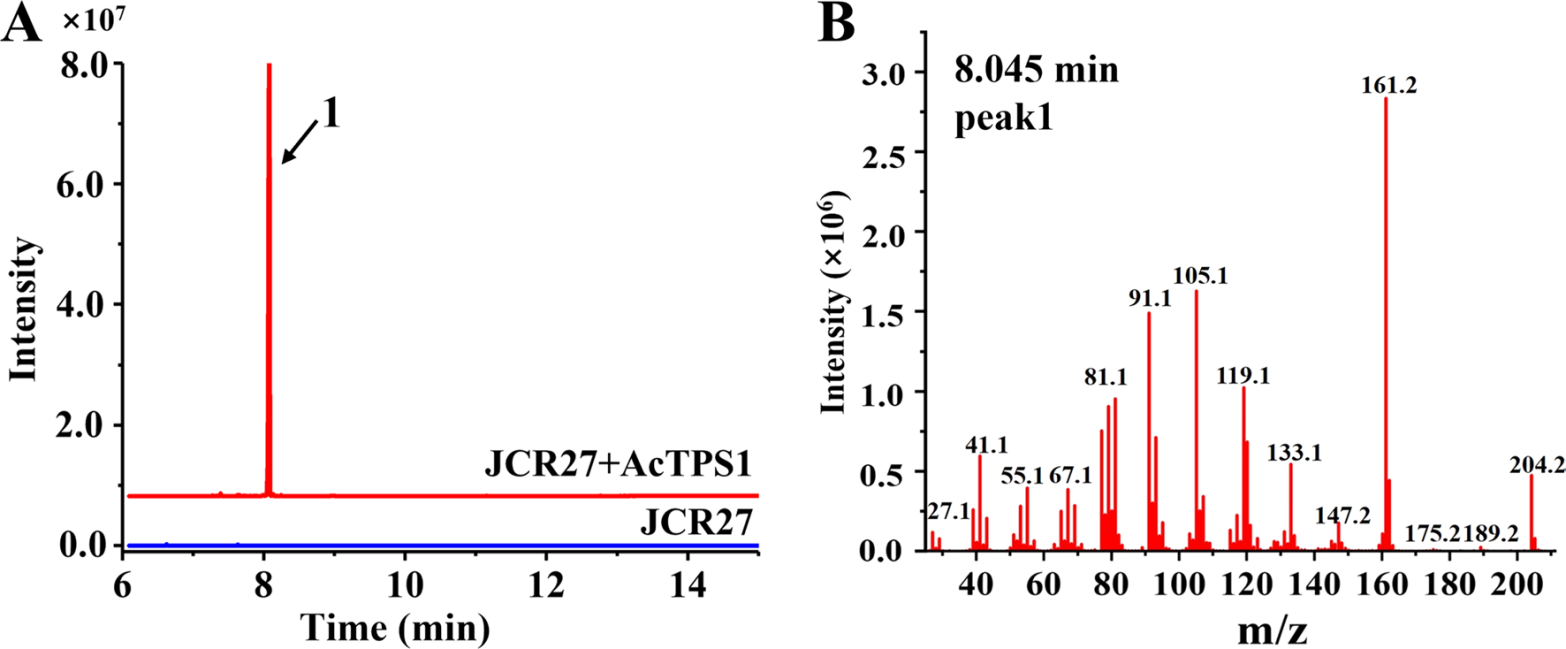
GC/MS analysis of volatile organic compounds produced by yeast transformants expressing AcTPS1*.* **A** GC-FID detection of AcTPS1 products in the yeast strain. Peak 1 represents the major product of AcTPS1 and is shown with a blank arrow. JCR27, the starting yeast strain used in this study. JCR27 + AcTPS1, AcTPS1 expressed in JCR27. **B** Detected by GC–MS, the major product of AcTPS1 is shown by the 204 m/z ion in the chromatographic trace

The fermentation results showed that except for AcTPS4, the remaining enzymes could produce sufficient products for GC–MS analysis. Yeast cultures expressing AcTPS1 and AcTPS5 accumulated one major sesquiterpene, accounting for > 98% and > 96% of total products detected, respectively (Fig. 3A, Additional file 1: Fig. S4). AcTPS2 produced multiple products and the major product was not a sesquiterpene. The second abundant product of AcTPS2 was β-farnesene according to the retention time and the mass peaks were consistent with those of the standard (Additional file 1: Fig. S5). AcTPS3 produced several compounds and the major volatile compound in AcTPS3 yeast strain cultures had a mass fragmentation pattern typical for a sesquiterpene with a parent ion at 204 m*/z* (Additional file 1: Fig. S6). The major product of AcTPS3 and AcTPS5 were not germacrene D, and they were unidentified since there were no related standards. The product of AcTPS1 is most likely germacrene D according to the retention time (8.045 min) and the mass peaks (Fig. 3), which were in line with the standard (Additional file 1: Fig. S7).

**Fig. 4:**
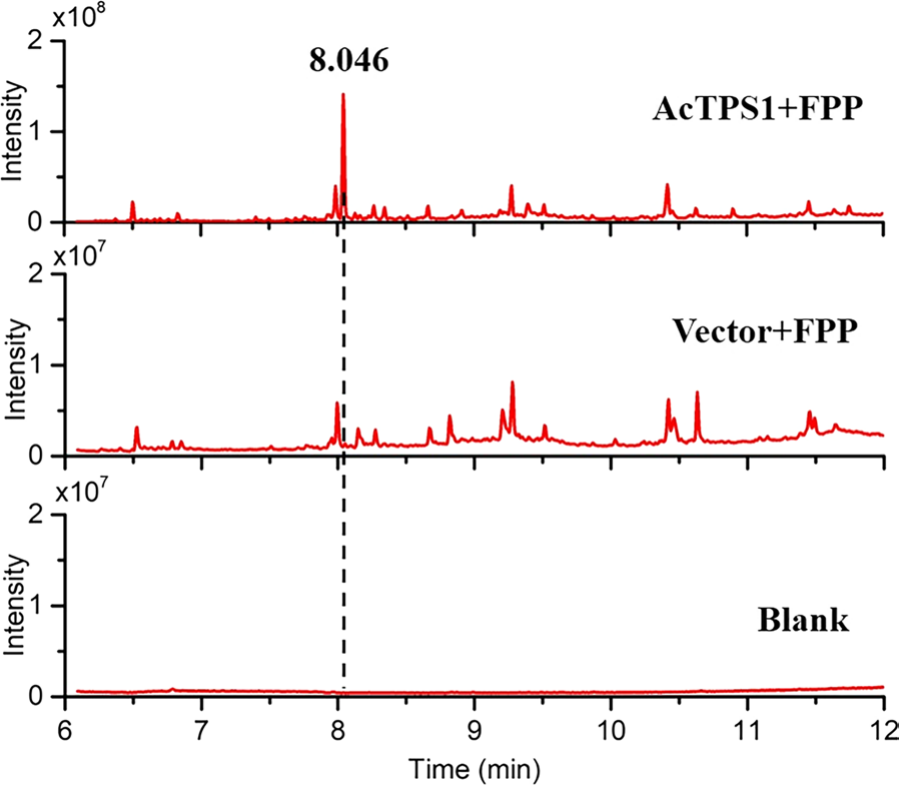
GC spectrum of the in vitro analysis of AcTPS1 enzyme function. The AcTPS1 enzymatic product is shown as a blank arrow. Blank, solvent hexane; Vector + FPP, pET-45b cell extracts reacting with the substrate FPP; AcTPS1 + FPP, AcTPS1 protein cell extracts reacting with the substrate FPP. AcTPS1 + FPP was concentrated tenfold for detection

### In vitro enzymatic reaction of AcTPS1 with the precursor FPP

For in vitro enzymatic analysis the recombinant AcTPS1 protein was expressed and purified. The plasmid pLJJ6 was constructed and transferred into *Escherichia coli Rosetta* to obtain the AcTPS1 protein expression strain LEc1. The weight of the recombinant AcTPS1 protein was close to the theoretical weight 41.03 KDa as shown by SDS–PAGE analysis (Additional file 1: Fig. S8). FPP was used as substrate in the in vitro enzymatic experiment to detect the catalytic activity of the AcTPS1 protein. The catalytic products were detected by GC–MS after 2 h catalytic reaction of the AcTPS1 protein or empty vector with FPP. The GC–MS results showed that a clear peak with the same retention time (8.046 min) as that observed in the in vivo experiments appeared in the AcTPS1 supplemented sample (Fig. 4). Moreover, the mass fragments of this peak were also exactly similar to the peak of the in vivo experiments (Additional file 1: Fig. S9).

To further confirm the structure of the AcTPS1 major product, the product was purified and _1_H and _13_C NMR spectrometry experiments were conducted. The results revealed that the _1_H and _13_C NMR chemical shifts (Additional file 1: Figs. S10, S11) were consistent with those of the germacrene D described in a previous report [28]. The specific rotation value of the AcTPS1 major product was ${\left[\mathrm{\alpha }\right]}_{D}^{20}$ -30.667 (c = 0.25, CHCl_3_); the specific rotation value of the (–)-germacrene D standard was ${\left[\mathrm{\alpha }\right]}_{D}^{20}$ -44.871 (c = 0.26, CHCl_3_) (Additional file 1: Table S5). Thus, AcTPS1 from *A. chrysogenum* was found to synthesize (–)-germacrene D for the first time.

### Comparing enzymes for high germacrene D titers in *Saccharomyces cerevisiae*

Knowing that sesquiterpene germacrene D has a variety of biological and structural functions, we decided to engineer *S. cerevisiae* to realize the high production of germacrene D. As terpene synthases are the bottlenecks in microbial sesquiterpene production [29, 30], we screened and compared the germacrene D titers of the different germacrene D synthases. In previous studies, many germacrene D synthases have been analyzed through in vitro assays. Some of germacrene D synthases displayed poor product specificity, producing large amounts of various byproducts (Additional file 1: Table S1). Therefore, those enzymes were excluded, and twenty germacrene D synthases that produced almost a single product were selected for the subsequent studies, as shown in red in Additional file 1: Table S1. Among these, the germacrene D synthases Sc19 and Sc11 from *Solidago canadensis*, PatTpsBF2 from *Pogostemon cablin*, AdGDS1 from *Actinidia deliciosa*, OvTPS3 from *Origanum vulgare*, ZpTPS2 from *Zanthoxylum piperitum*, DcTPS7 from *Daucus carota* L produced germacrene D as approximately 95% of their product profiles, as described in the literatures (Additional file 1: Table S1).

**Fig. 5:**
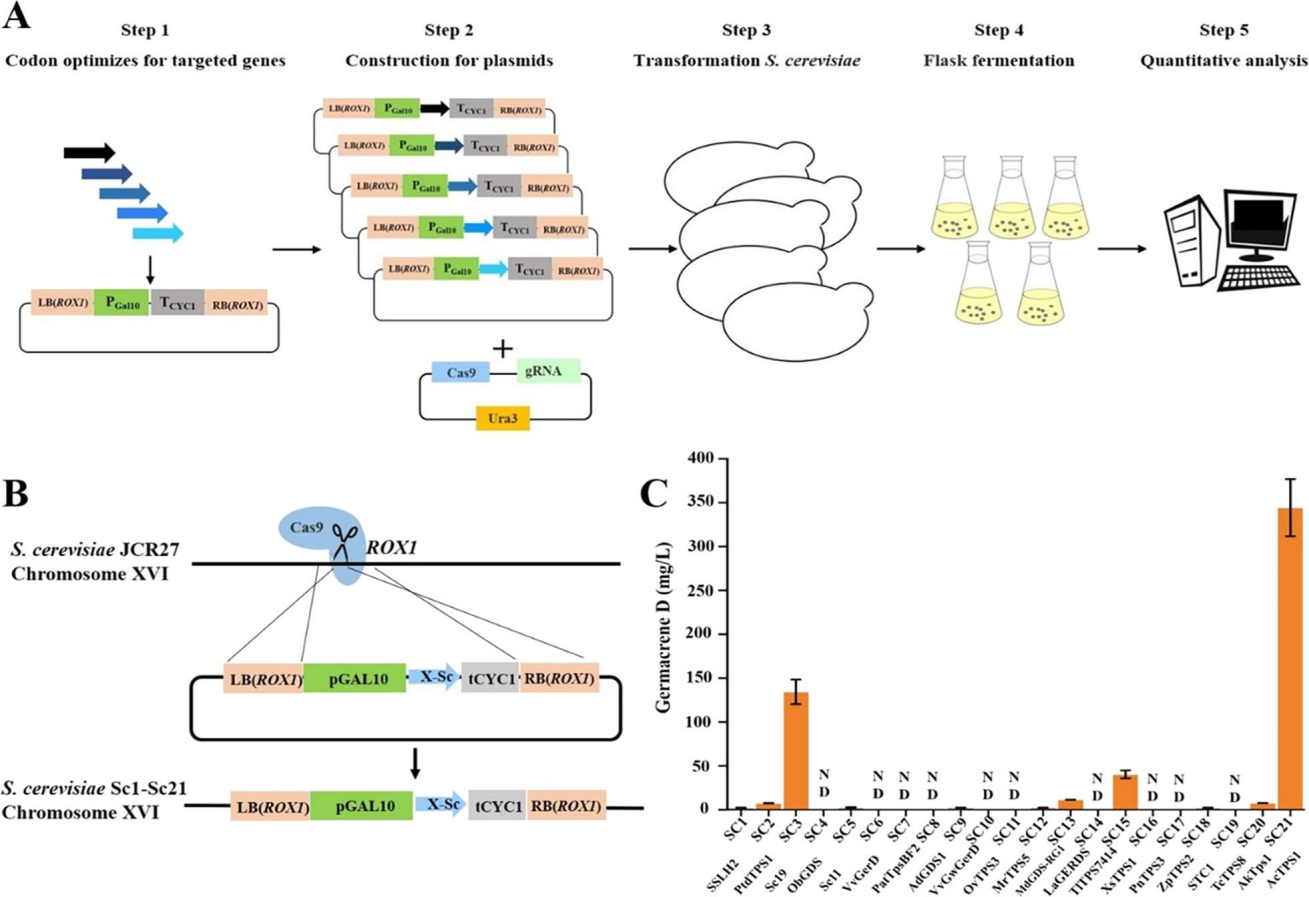
Comparing germacrene D synthases for high germacrene D production in *S. cerevisiae*. **A** Schematic diagram of comparing germacrene D production in *S. cerevisiae*. Transformation, flask fermentation and quantitative analysis were performed as described in “Methods” (B) Strategy for the construction of SC1-SC21 via homologous recombination. JCR27, the starting strain; SC1-SC21, yeast strains expressing different germacrene D synthases; *ROX1*, the insertion site for exogenous fragments; LB (*ROX1*), the upstream sequence of rox1; RB (*ROX1*), the downstream sequence of *ROX1*; tCYC1, the terminator of CYC1; X-Sc, the germacrene D gene optimized for *S. cerevisiae*; pGAL1pGAL10, the promoter of *GAL1* and *GAL10*. **B** The production of different germacrene D producing strains. Germacrene D levels were determined after 72 h fermentation in YPDH medium as described in the “Methods”. Error bars represent standard deviations from three independent experiments. ND, not detected. SSLH2 et al., the names of germacrene D synthases from different organisms detailed in the text

We employed the terpene precursor-producing strain JCR27 as the platform. The *GAL1* promoter was used to express germacrene D synthases. The gRNA-tRNA array for CRISPR–Cas9 (GTR-CRISPR) was modified and applied for multiplexed engineering of *S. cerevisiae* [31] (Additional file 1: Fig. S12). Briefly, as shown in Fig. 5A, we synthesized the *S. cerevisiae* codon-optimized version of the abovementioned twenty germacrene D synthases, as shown in red in Additional file 1: Table S1, then put these germacrene D synthase gene coding sequences and *Actps1* under the control of *GAL1* promoter to assemble pLJJ7-PLJJ27 plasmid. The plasmid pLJJ7-pLJJ27, together with pLJJ28, was transformed into JCR27 to obtain the engineered strains SC1-SC21, in which germacrene D synthase expression cassettes replaced *ROX1* (Fig. 5B). Three replicates of each strain were tested with shake-flask fermentation in 50 mL YPDGH medium, and the products of SC1-SC21 were detected and quantified by GC–MS.

Additional file 1: Figure. S13 and S14 show the results of germacrene D production of strain SC1-SC21. According to the heterologous expression results, we found that half of these enzymes exhibited low activities and did not produce detectable levels of products, including SC4, SC6, SC7, SC8, SC10, SC11, SC14, SC16, SC17 and SC19 (Additional file 1: Fig. S13, S14). Only eleven enzymes could produce sufficient products for GC–MS analysis (Fig. 5C). Proteins Sc11 (expressed in strain SC5) and Sc19 (expressed in strain SC3) were both isolated from *Solidago canadensis* and identified as ( +)-germacrene D synthase, (–)-germacrene D synthase respectively [32]. The production of (–)-germacrene D reached to 134.3 ± 14.1 mg/L in SC3, which was much higher than that in SC5, indicating that Sc19 is a more robust enzyme. In addition, XsTPS1 cloned from *Xanthium strumarium* produced 40.3 ± 4.6 mg/L germacrene D, but the absolute configuration was unknown [33]. The (–)-germacrene D production of SC21 expressed with AcTPS1 was 344.4 ± 32.6 mg/L, representing the highest production among all the tested enzymes (Additional file 1: Table S6). These results demonstrated that most germacrene D synthases displayed lower activity in heterologous expression. AcTPS1 produced the highest production of germacrene D with high selectivity. It was reported that ( +)-δ-cadinene synthase, which is a “high-fidelity” sesquiterpene cyclase as it generates ( +)-δ-cadinene nearly exclusively (> 98%), also contains two characteristic aspartate-rich motifs, D_307_DTYD_311_ and D_451_DVAE_455_ [34]. In the future more structure research of AcTPS1 may help elucidate the catalytic mechanism of the two DDXXD domains. These results suggested that AcTPS1 from *A. chrysogenum* exhibited extraordinarily high activity and high selectivity, which can be an excellent biosynthetic element for the next metabolic engineering.

### Engineering yeast to overproduce (–)-germacrene D

**Fig. 6:**
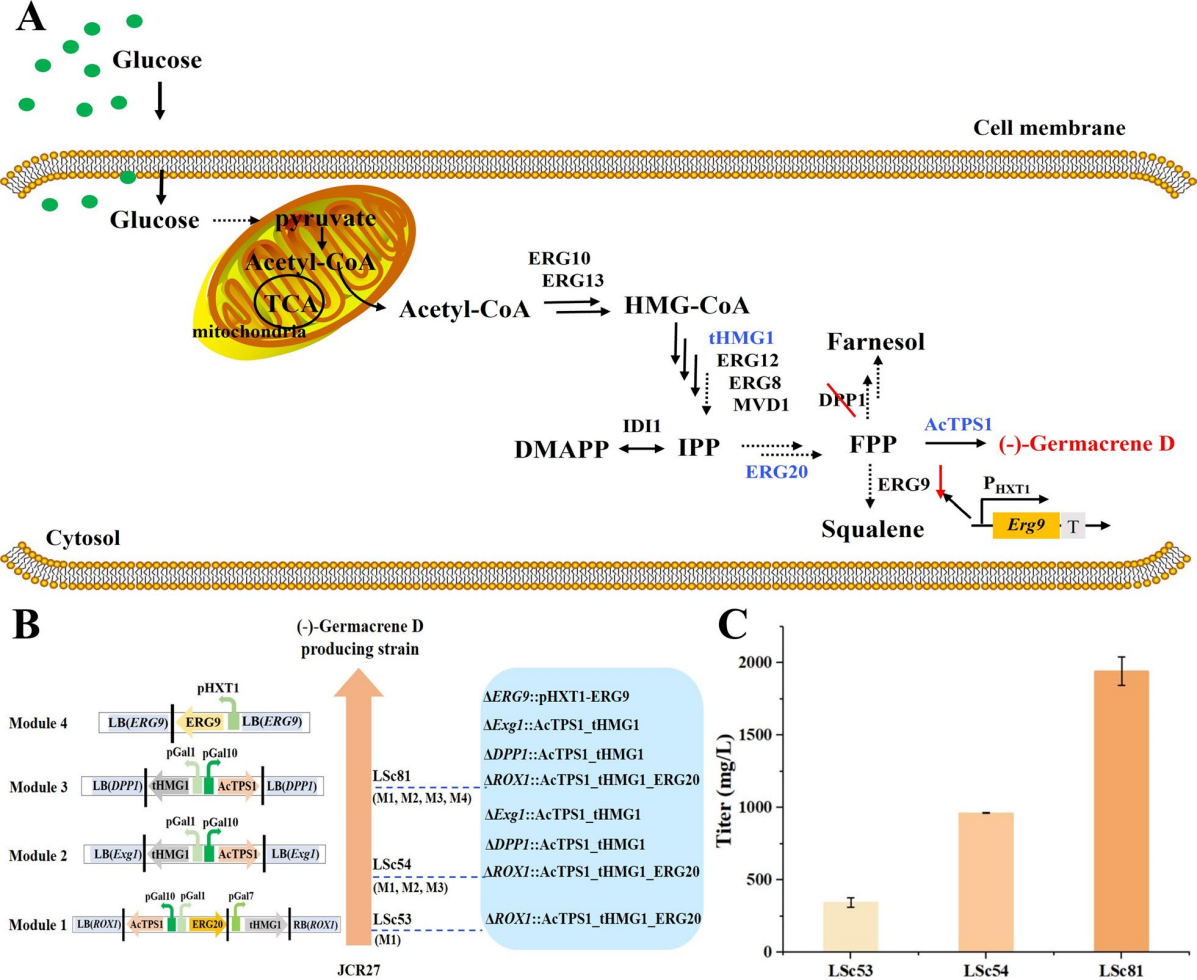
Titer of (–)-germacrene D in engineered yeast strains in shake-flask fermentation. **A** Biosynthesis pathways for (–)-germacrene D in engineered yeast. *ERG10* acetyl-CoA C-acetyltransferase, *ERG13* 3-hydroxy-3-methylglutaryl coenzyme A synthase, *tHMG1* truncated 3-hydroxy-3-methylglutaryl-coenzyme A reductase 1, *ERG8* phosphomevalonate kinase, *ERG12* mevalonate kinase, *MVD1* mevalonate diphosphate decarboxylase 1, *IDI1* isopentenyl-diphosphate delta isomerase 1, *ERG20* farnesyl pyrophosphate synthetase, *AcTPS1* (–)-germacrene D synthase. Overexpressed enzymes are shown in blue. Red slash, gene knockout; Red downward arrow, downregulated gene expression. **B** The engineered yeast strains integrated with gene modules. These expression modules were integrated into the chromosomal sites of *ROX1* (module 1, chromosome XIV), *Exg1* (module 2, chromosome XII), *DPP1* (module 3, chromosome IV), and *ERG9* (module 4, chromosome VIII). The engineered strain LSc53 contained expression module 1 (abbreviated as ‘M1’). The engineered strain LSc54 contained the expression module 1 (‘M1’), module 2 (‘M2’) and module 3 (‘M3’). The engineered strain LSc81 contained the expression module 1 (‘M1’), module 2 (‘M2’), module 3 (‘M3’) and module 4 (‘M4’). (C) The production of different engineered stains. Germacrene D levels were determined after 72 h fermentation in YPDH medium as described in “Methods”. Error bars represent standard deviations from three independent experiments

After obtaining the best performed (–)-germacrene D producing enzyme (AcTPS1), we set out to improve the production of (–)-germacrene D in *S. cerevisiae* via metabolic engineering. We integrated *Actps1* into the terpene precursor-enhancing strain JCR27 and iteratively engineered the mutant strain to fulfill the (–)-germacrene D production potential by overexpressing the rate-limiting enzyme and repressing the competitive pathway (Fig. 6A).

The GAL promoters have been proven to be highly effective in sesquiterpene production, and the strength of *GAL1* and *GAL10* promoters was found to rapidly improve after glucose was exhausted [35]. Thus, all the heterologously expressed genes were placed under the control of the GAL promoters, including *GAL1*, *GAL7* and *GAL10* promoters, to achieve high expression of (–)-germacrene D. First, the synthetic gene *Actps1* was combined with *ERG20 and tHMG1* using GAL promoters. Then this construct (‘M1’ in Fig. 6B) was integrated into the *ROX1* gene locus, which encodes a negative regulator of the MVA pathway, to generate the LSc53 mutant strain [36] (Fig. 6B). The result of the fermentation of LSc53 showed that coupled with the overexpression of *tHMG1* and *ERG20*, overexpressed *AcTPS1* achieved 376.2 ± 30.2 mg/L (–)-germacrene D in shake-flask conditions (Fig. 6C). These results indicated that the strategy of overexpressing genes with GAL promoters was efficient to increase the supply of FPP and improve the (–)-germacrene D production.

Increasing the gene copy numbers to strengthen the target metabolic pathway flux is regarded as an effective strategy for improving target production [37]. In addition, overexpression of *tHMG1* had a good effect on the production of terpenes in *S. cerevisiae* in a large number of studies [18]. Therefore, two additional *tHMG1* and *Actps1* genes were combined with GAL promoters to generate another two constructs (‘M2 and M3’ in Fig. 6B) for subsequent engineering. It was reported in a previous study that *DPP1* were accounted for the most hydrolytic activities, and the loss of FPP could be decreased by deleting *DPP1* [38]. Therefore, the two constructs were added to the *DPP1* gene locus (encoding phosphatidate phosphatase) and *Exg1* gene locus (encoding glucan 1,3-beta-glucosidase) of LSc53 to generate the LSc54 mutant strain. The shake-flask fermentation results showed that the production of (–)-germacrene D was significantly increased to 963.8 ± 3.1 mg/L in LSc54, which was approximately 2.6 times higher than that in the original LSc53 strain (Fig. 6C). These results suggested that the expression levels of AcTPS1 and tHMG1 were vital factors affecting target compound synthesis.

Squalene synthase, encoded by *ERG9*, is one of the key enzymes involved in sterol formation and has become a key target for increasing terpene production. It was reported that downregulating the *ERG9* gene by using the P_HXT1_ promoter could sharply increase α-santalene production by increasing the FPP pool [39]. To further improve the (–)-germacrene D titer, *ERG9* was constructed under the control of the glucose-regulated promoter P_HXT1_ to generate an additional module (‘M4’ in Fig. 6B). The construct was integrated into the *ERG9* gene locus to substitute the *ERG9* endogenous promoter with the *HXT1* promoter in LSc53, generating the mutant strain LSc81 (Fig. 6B). The results showed that the (–)-germacrene D titer increased to 1943.0 ± 97.7 mg/L during shake-flask fermentation, which was two times higher than that in LSc54 (Fig. 6C). Consequently, we obtained a high producing (–)-germacrene D strain via metabolic engineering of *S. cerevisiae*.

### Fed-batch fermentation for high-level (–)-germacrene D titers

**Fig. 7:**
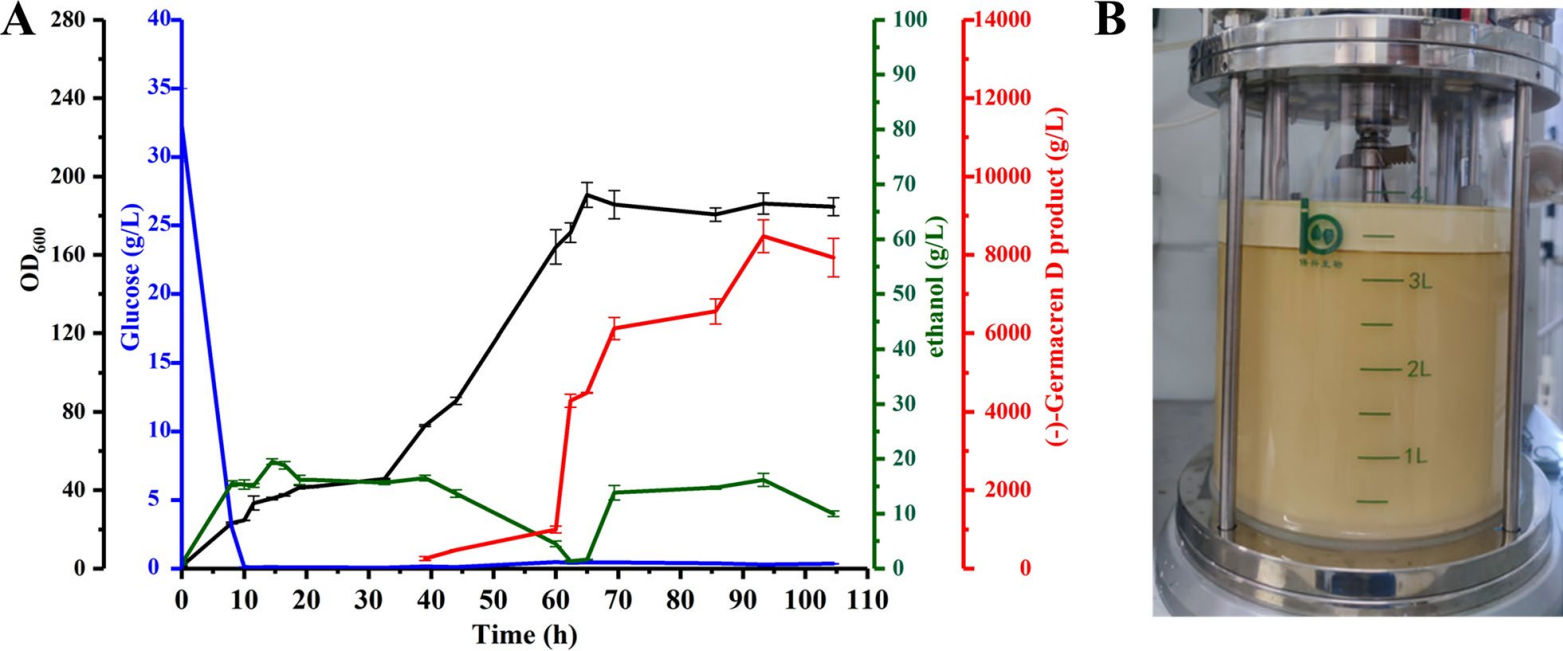
High-density fermentation of strain LSc81 for (–)-germacrene D production. **A** Results of fermentation in a 5-L bioreactor. (–)-Germacrene D production, cell growth, and residual concentrations of glucose and ethanol during fed-batch fermentation were measured. Error bars indicate the standard deviations of three replicates. **B** Fermentation broth in the 5-L bioreactor. The upper oil phase contained isopropyl myristate and (–)-germacrene D

To evaluate the production property of the engineered strain LSc81, fed-batch fermentation was carried out with a 5-L bioreactor using the synthetic medium CSM. In consideration of the O_2_ requirements during the rapid growth stage of the engineered strain, oxygen supply was increased by changing the ventilation from 0.8 to 2 (air volume/culture volume/min, vvm) and adjusting the stirring rate to 200–500 rpm. To achieve a balance between strain growth and product accumulation, two-stage fermentation was employed. As shown in Fig. 7, the biomass of LSc81 increased constantly after the second seed was incubated in the 5-L bioreactor. The residual amount of glucose began to decrease from the incubation and reached 0.1 g/L at 10 h. Then the feeding solution I was added into the bioreactor at an appropriate flow rate. The glucose concentration was kept around 1 g/L to maintain cell growth. The growth curve flattened until 32.5 h, probably because of insufficient nutrition to support the cell growth. Then 400 mL YPD was added to the cultures, and the cells began to grow rapidly, reaching an OD_600_ of 154 at 60 h. Ethanol began to decrease at 16.5 h and reached 3.4 g/L at 60 h. Then feeding solution I was replaced with feeding solution II (ethanol) for product accumulation, and ethanol was maintained at an appropriate level until the end of fermentation. The fermentation cycle of the 5-L bioreactor was approximately 110 h, and the (–)-germacrene D production reached 7.9 g/L. This result demonstrates the excellent high production performance of the engineered strain LSc81.

However the full potential of LSc81 productivity was not realized during fed-batch fermentation compared with the impressive production observed with shake-flask fermentation. It was reported that the production of bicyclic sesquiterpene (–)-eremophilene reached 708.5 mg/L in shake-flask fermentation, and a titer of 34.6 g/L was finally achieved, benefitting from the high titer in fed-batch fermentation [23]. For our strain LSc81, the final OD (reaching the OD_600_ of 184) was significantly lower than that of the reported strain JHM5 [23]. Prototrophic strains can achieve high-density fermentation, and the target production could be further improved [30]. Thus complementing the current mutant strain LSc81 with auxotrophic markers may improve the present productivity using fed-batch fermentation. Nevertheless the titer of (–)-germacrene D produced by LSc81 was the highest production at the laboratory level in this study.

## Conclusions

In this study, high production of (–)-germacrene D was achieved through metabolic engineering of *S. cerevisiae* using CRISPR–Cas9 genome editing technology. A new (–)-germacrene D synthase was identified from *A. chrysogenum* and introduced into a terpene precursor-enhancing yeast strain to achieve (–)-germacrene D biosynthesis. By combining the expression of three copies of AcTPS1 with various MVA pathway engineering approaches, the highest (–)-germacrene D titer (7.9 g/L) was achieved in fed-batch fermentation in a 5-L bioreactor, which was the highest production reported thus far. In conclusion, our work presented an attractive approach that can serve as a reference for achieving high production of sesquiterpenes. 

## Supplementary Information

**Additional file 1: Table S1**. Germacrene D synthases from different organisms. **Table S2**. Strains and plasmids used in this study. **Table S3**. Primers used in this study. **Table S4**. Terpene synthases used for phylogenetic analysis. **Table S5**. The specific rotation of the product of AcTPS1 and (–)-Germacrene D standard. **Table S6**. The germacrene D production in different engineered yeaststrains. **Fig. S1**. Amino acids alignment of the sesquiterpene synthases from Acremonium chrysogenum. **Fig. S2**. Amino acids alignment of STC1, AcTPS1 and AcTPS3. **Fig. S3**. The transcription of Actps1to Actps5 inA. chrysogenum. **Fig. S4**. GC spectrum and the corresponding Mass spectra of sesquiterpenes biosynthesized by AcTPS5. **Fig. S5**. GC spectrum and the corresponding Mass spectra of sesquiterpenes biosynthesized by AcTPS2. **Fig. S6**. GC spectrum and the corresponding Mass spectra of sesquiterpenes biosynthesized by AcTPS3. **Fig. S7**. (–)-Germacrene D standard mass spectrum detected by GC-MS. **Fig. S8**. Purified AcTPS1 protein from recombinant Escherichia coli. **Fig. S9**. The mass spectra of the AcTPS1 enzymatic product. **Fig. S10**. 13C NMR spectra of (–)-Germacrene D (400 MHz, CDCl3).**Fig. S11**. 1H NMR spectrum of (–)-Germacrene D 30 (400 MHz, CDCl3). **Fig. S12**. The schematic of gene editing with CRISPR/Cas9 system mediated by a recyclable gRNA plasmid. **Fig. S13**. GC spectrum of SC1-SC12. **Fig. S14**. GC spectrum of SC13-SC21, JCR27 and germacrene D standard.

## Data Availability

All data for this study are included in this published article and its additional file.

## Abbreviations

FPP: Farnesyl diphosphate
BGCs: Biosynthetic gene clusters
CPC: Cephalosporin C
MVA: Mevalonate pathway
IPP: Isopentenyl diphosphate
DMAPP: Dimethylallyl pyrophosphate
FPPS: Farnesyl diphosphate synthase
GC–MS: Gas chromatography-mass spectrometry
aa: Amino acid
( +)-δ-Cadinene synthase: DCS
