# Supplementary material for: Identification of the sesquiterpene synthase AcTPS1 and high production of (–)-germacrene D in metabolically engineered Saccharomyces cerevisiae
Source: Microb Cell Fact. 2022 May 18;21:89. doi: 10.1186/s12934-022-01814-4 (PMC9115970; doi:10.1186/s12934-022-01814-4)
Supplement: Supplementary file 1 — Additional file 1: Table S1. Germacrene D synthases from different organisms. Table S2. Strains and plasmids used in this study. Table S3. Primers used in this study. Table S4. Terpene synthases used for phylogenetic analysis. Table S5. The specific rotation of the product of AcTPS1 and (–)-Germacrene D standard. Table S6. The germacrene D production in different engineered yeaststrains. Fig. S1. Amino acids alignment of the sesquiterpene synthases from Acremonium chrysogenum. Fig. S2. Amino acids alignment of STC1, AcTPS1 and AcTPS3. Fig. S3. The transcription of Actps1to Actps5 inA. chrysogenum. Fig. S4. GC spectrum and the corresponding Mass spectra of sesquiterpenes biosynthesized by AcTPS5. Fig. S5. GC spectrum and the corresponding Mass spectra of sesquiterpenes biosynthesized by AcTPS2. Fig. S6. GC spectrum and the corresponding Mass spectra of sesquiterpenes biosynthesized by AcTPS3. Fig. S7. (–)-Germacrene D standard mass spectrum detected by GC-MS. Fig. S8. Purified AcTPS1 protein from recombinant Escherichia coli. Fig. S9. The mass spectra of the AcTPS1 enzymatic product. Fig. S10. 13C NMR spectra of (–)-Germacrene D (400 MHz, CDCl3).Fig. S11. 1H NMR spectrum of (–)-Germacrene D 30 (400 MHz, CDCl3). Fig. S12. The schematic of gene editing with CRISPR/Cas9 system mediated by a recyclable gRNA plasmid. Fig. S13. GC spectrum of SC1-SC12. Fig. S14. GC spectrum of SC13-SC21, JCR27 and germacrene D standard. [file 12934_2022_1814_MOESM1_ESM.docx]

**Additional file 1**

**Identification of the sesquiterpene synthase AcTPS1 and high production of (-)-germacrene D in metabolically engineered *Saccharomyces cerevisiae***

Jiajia Liu^1#^, Chang chen^1#^, Xiukun Wan^1^, Ge Yao^1^, Shaoheng Bao^1^, Fuli Wang^1^, Kang Wang^1^, Tianyu Song^1^, Penggang Han^1^ and Hui Jiang^1^*

^1^ State Key Laboratory of NBC Protection for Civilian, Academy of Military Sciences, Beijing, 102205, People’s Republic of China

^#^ These authors contributed equally to this work, ^*^ Correspondence

Email addresses of authors:

Jiajia Liu, Email: [jiajialiu0802@163.com](mailto:jiajialiu0802@163.com)

Chang Chen, Email: [chenchang9@qq.com](mailto:chenchang9@qq.com)

Xiukun Wan, Email: [xiukunwan@126.com](mailto:xiukunwan@126.com)

Ge Yao, Email: [bzyaoge@163.com](mailto:bzyaoge@163.com)

Shaoheng Bao, Email: [603716673@qq.com](file:///C:\Users\jiajia\Desktop\AcTPS1%20投稿\%20603716673@qq.com)

Fuli Wang, Email: [88082136@qq.com](file:///C:\Users\jiajia\Desktop\AcTPS1%20投稿\88082136@qq.com)

Kang Wang, Email: [yiyongjun1949@163.com](file:///C:\Users\jiajia\Desktop\AcTPS1%20投稿\yiyongjun1949@163.com)

Tianyu Song, Email: [songtianyu90@foxmail.com](mailto:songtianyu90@foxmail.com)

Penggang Han, Email: [hanpeng1021@163.com](file:///C:\Users\jiajia\Desktop\AcTPS1%20投稿\hanpeng1021@163.com)

Hui Jiang, Email: [ylplkmc@163.com](mailto:ylplkmc@163.com)

**Content**

**Table S1** Germacrene D synthases from different organisms.

**Table S2** Strains and plasmids used in this study.

**Table S3** Primers used in this study.

**Table S4** Terpene synthases used for phylogenetic analysis.

**Table S5** The specific rotation of the product of AcTPS1 and (-)-Germacrene D standard.

**Table S6** The germacrene D production in different engineered yeast strains.

**Fig. S1** Amino acids alignment of the sesquiterpene synthases from *Acremonium chrysogenum*.

**Fig. S2** Amino acids alignment of STC1, AcTPS1 and AcTPS3.

**Fig. S3** The transcription of *Actps1*to *Actps5* in *A. chrysogenum.*

**Fig.** **S4** GC spectrum and the corresponding Mass spectra of sesquiterpenes biosynthesized by AcTPS5.

**Fig. S5** GC spectrum and the corresponding Mass spectra of sesquiterpenes biosynthesized by AcTPS2.

**Fig. S6** GC spectrum and the corresponding Mass spectra of sesquiterpenes biosynthesized by AcTPS3.

**Fig. S7** (-)-Germacrene D standard mass spectrum detected by GC-MS.

**Fig. S8** Purified AcTPS1 protein from recombinant *Escherichia coli*.

**Fig. S9** The mass spectra of the AcTPS1 enzymatic product.

**Fig. S10** ^13^C NMR spectra of (-)-Germacrene D (400 MHz, CDCl_3_).

**Fig. S11** ^1^H NMR spectrum of (-)-Germacrene D (400 MHz, CDCl_3_).

**Fig. S12** The schematic of gene editing with CRISPR/Cas9 system mediated by a recyclable gRNA plasmid.

**Fig. S13** GC spectrum of SC1-SC12.

**Fig. S14** GC spectrum of SC13-SC21, JCR27 and germacrene D standard.

**Table S1** Germacrene D synthases from different organisms.

| **ID No.** | **Protein** | **Accession No.** | **Organism** | **Strain** | **Reference** |
| --- | --- | --- | --- | --- | --- |
| 1 | SSLH2 | AF279456.1 | *Lycopersicon esculentum* | SC1 | [1] |
| 2 | Can’t find | MG673512.1 | *Rosa Hybrida* | — | [2] |
| 3 | SCO6073 | NP_630182.1 | *Streptomyces coelicolor A3(2)* | — | [3] |
| 4 | PtdTPS1 | AAR99061.1 | *Populus trichocarpa X Populus deltoides* | SC2 | [4] |
| 5 | Sc19 | AJ583448.1 | *Solidago canadensis* | SC3 | [5] |
| 6 | ObGDS | AAV63786.1 | *Ocimum basilicum* | SC4 | [6] |
| 7 | Sc11 | AJ583447.1 | *Solidago canadensis* | SC5 | [5] |
| 8 | VvGerD | AAS66357.1 | *Vitis vinifera L.* | SC6 | [7] |
| 9 | PatTpsBF2 | AAS86320.1 | *Pogostemon cablin* | SC7 | [8] |
| 10 | PatTpsB15 | AAS86322.1 | *Pogostemon cablin* | — | [8] |
| 11 | unknown | AY860846.1 | *Zingiber oYcinale* | — | [9] |
| 12 | AdGDS1 | AY789791.1 | *Actinidia deliciosa* | SC8 | [10] |
| 13 | VvGwGerD | HM807377.1 | *Vitis vinifera* | SC9 | [11] |
| 14 | OvTPS3 | ADK73619.1 | *Origanum vulgare* | SC10 | [12] |
| 15 | PtTS1 | AEI52901.1 | *Populus trichocarpa* | — | [13] |
| 16 | VMPSTS | ABX57720.1 | *Orchid Fragrance* | — | [14] |
| 17 | VoTPS1 | J9RLZ7.1 | *Valeriana officinalis* | — | [15] |
| 18 | MrTPS5 | AFM43738.1 | *Matricaria chamomilla* | SC11 | [16] |
| 19 | MdGDS-RG1 | JX848730.1 | *Malus domestica* | SC12 | [17] |
| 20 | OrTPS1 | AIJ00876.1 | *Oryza rufipogon* | — | [18] |
| 21 | LaGERDS | U3LVL5.1 | *Lavandula angustifolia* | SC13 | [19] |
| 22 | TlTPS7414 | KR822706.1 | *Nicotiana benthamiana* | SC14 | [20] |
| 23 | XsTPS1 | KT317705.1 | *Xanthium strumarium* | SC15 | [21] |
| 24 | PnTPS3 | MF104556.1 | *Black pepper* | SC16 | [22] |
| 25 | ZpTPS2 | BBD88589.1 | *Zanthoxylum piperitum* | SC17 | [23] |
| 26 | STC1 | XM_023573743.1 | *Fusarium fujikuroi* | SC18 | [24] |
| 27 | VvShirazTPS07 | NP_001268063.1 | *Vitis vinifera L.* | — | [25] |
| 28 | SgSTPS2 | Can’t find | *Sindora Glabra* | — | [26] |
| 29 | AscTps2a | LC387450.1 | *Chengiopanax sciadophylloides* | — | [27] |
| 30 | TcTPS8 | QGN65614.1 | *Taiwania cryptomerioides* | SC19 | [28] |
| 31 | PvTPS55 | Can’t find | *Panicum virgatum L.* | — | [29] |
| 32 | McGDS2 | Unigene24738 | *Matricaria chamomilla L* | — | [30] |
| 33 | DcTPS7 | Can’t find | *Daucus carota L.* | SC20 | [31] |
| 34 | AkTps1 | LC506578.1 | *Angelica keiskei* | — | [32] |
| 35 | FcTPS1 | MT086603.1 | *Ficus carica* | — | [33] |
| 36 | CaSTS2 | Can’t find | *Celastrus angulatus* | — | [34] |
| 37 | ZaTPS1 | UJH94377.1 | *Zanthoxylum ailanthoides* | — | [35] |
| 38 | ZaTPS2 | UJH94378.1 | *Zanthoxylum ailanthoides* | — | [35] |
| 39 | ZaTPS3 | UJH94379.1 | *Zanthoxylum ailanthoides* | — | [35] |
| 40 | Cop1 | XP_001832573.1 | *Coprinus cinereus* | — | [36] |
| 41 | Cop3 | XP_001832925.1 | *Coprinus cinereus* | — | [36] |
| 42 | Cop4 | XP_001836356.1 | *Coprinus cinereus* | — | [36] |

**Table S2** Strains and plasmids used in this study.

| **Strains or plasmids** | **Characteristics** | **Source** |
| --- | --- | --- |
| **Strains** | | |
| JCR27 | ChrXII-2Δ:: *HygR*-P_TEF1_-*Cas9*; ChrXI-3Δ:: P_GAL1_-*ERG8*, P_GAL10_-*tHMG1*, P_GAL7_-*ERG12*; ChrX-3Δ:: P_GAL1_-*ERG13*, P_GAL10_-*tHMG1*; ChrXII-4Δ:: P*GAL1*-*IDI1*, P*GAL10*-*ERG10*, P*GAL7*-*MVD1* | [37] |
| LSc1 | pGAL1-*Actps1*, free replicating plasmid | This study |
| LSc2 | pGAL1-*Actps2*, free replicating plasmid | This study |
| LSc3 | pGAL1-*Actps3*, free replicating plasmid | This study |
| LSc4 | pGAL1-*Actps4*, free replicating plasmid | This study |
| LSc5 | pGAL1-*Actps5*, free replicating plasmid | This study |
| SC1 | *rox1*Δ:: P_GAL1_-*SSLH2* | This study |
| SC2 | *rox1*Δ:: P_GAL1_-*PtdTPS1* | This study |
| SC3 | *rox1*Δ:: P_GAL1_-*Sc19* | This study |
| SC4 | *rox1*Δ:: P_GAL1_-*ObGDS* | This study |
| SC5 | *rox1*Δ:: P_GAL1_-*Sc11* | This study |
| SC6 | *rox1*Δ:: P_GAL1_-*VvGerD* | This study |
| SC7 | *rox1*Δ:: P_GAL1_-*PatTpsBF2* | This study |
| SC8 | *rox1*Δ:: P_GAL1_-*AdGDS1* | This study |
| SC9 | *rox1*Δ:: P_GAL1_-*VvGwGerD* | This study |
| SC10 | *rox1*Δ:: P_GAL1_-*OvTPS3* | This study |
| SC11 | *rox1*Δ:: P_GAL1_-*MrTPS5* | This study |
| SC12 | *rox1*Δ:: P_GAL1_-*MdGDS-RG1* | This study |
| SC13 | *rox1*Δ:: P_GAL1_-*LaGERDS* | This study |
| SC14 | *rox1*Δ:: P_GAL1_-*TlTPS7414* | This study |
| SC15 | *rox1*Δ:: P_GAL1_-*XsTPS1* | This study |
| SC16 | *rox1*Δ:: P_GAL1_-*PnTPS3* | This study |
| SC17 | *rox1*Δ:: P_GAL1_-*ZpTPS2* | This study |
| SC18 | *rox1*Δ:: P_GAL1_-*STC1* | This study |
| SC19 | *rox1*Δ:: P_GAL1_-*TcTPS8* | This study |
| SC20 | *rox1*Δ:: P_GAL1_-*DcTPS7* | This study |
| SC21 | *rox1*Δ:: P_GAL1_-*Actps1* | This study |
| LSc53 | *rox1*Δ:: P_GAL10_-*Actps1*, P_GAL1_-*tHMG1,* P_GAL7_-*ERG20* | This study |
| LSc54 | *rox1*Δ:: P_GAL10_-*Actps1*, P_GAL1_-*tHMG1,* P_GAL7_-*ERG20*; *exg1*Δ:: P_GAL1_-*Actps1*, P_GAL10_-*tHMG1*; *dpp1*Δ:: P_GAL1_-*Actps1*, P_GAL10_-*tHMG1* | This study |
| LSc81 | *rox1*Δ:: P_GAL10_-*Actps1*, P_GAL1_-*tHMG1,* P_GAL7_-*ERG20*; *exg1*Δ:: P_GAL1_-*Actps1*, P_GAL10_-*tHMG1*; *dpp1*Δ:: P_GAL1_-*Actps1*, P_GAL10_-*tHMG1*; *ERG9*Δ:: P_HXT1_-*ERG9* | This study |
| *Escherichia coli* DH5α | Used for routine cloning | Gibco BRL |
| *Escherichia coli Rosseta* | Used for protein expression | TransGen |
| LEc1 | T7-*Actps1*, AcTPS1 protein expression strain | This study |
| **Plasmids** | | |
| pRS462 | Routine cloning and expressing vector, 2 μ origin, Amp^R^, *URA* | this lab |
| pEASY-Blunt | Routing cloning vector | TransGen |
| pET-45b | Protein expression vector | TransGen |
| pCas | Expressing Cas9 and gRNA vector for genome editing | [38] |
| SgRNA | contain the sequences of tRNA and gRNA scaffold | [38] |
| KlURA3 | contain the truncated URA3 | [38] |
| pLJJ1 | pRS426, pGAL1-*Actps1*, *URA*, AmpR | This study |
| pLJJ2 | pRS426, pGAL1-*Actps2*, *URA*, AmpR | This study |
| pLJJ3 | pRS426, pGAL1-*Actps3*, *URA*, AmpR | This study |
| pLJJ4 | pRS426, pGAL1-*Actps4*, *URA*, AmpR | This study |
| pLJJ5 | pRS426, pGAL1-*Actps5*, *URA*, AmpR | This study |
| pLJJ6 | pET-45b, T7-*Actps1*, AmpR | This study |
| pLJJ7 | Blunt, pGAL1- *SSLH2*, AmpR | This study |
| pLJJ8 | Blunt, pGAL1- *PtdTPS1*, AmpR | This study |
| pLJJ9 | Blunt, pGAL1- *Sc19*, AmpR | This study |
| pLJJ10 | Blunt, pGAL1- *ObGDS*, AmpR | This study |
| pLJJ11 | Blunt, pGAL1- *Sc11*, AmpR | This study |
| pLJJ12 | Blunt, pGAL1- *VvGerD*, AmpR | This study |
| pLJJ13 | Blunt, pGAL1- *PatTpsBF2*, AmpR | This study |
| pLJJ14 | Blunt, pGAL1- *AdGDS1*, AmpR | This study |
| pLJJ15 | Blunt, pGAL1- *VvGwGerD*, AmpR | This study |
| pLJJ16 | Blunt, pGAL1- *OvTPS3*, AmpR | This study |
| pLJJ17 | Blunt, pGAL1- *MrTPS5*, AmpR | This study |
| pLJJ18 | Blunt, pGAL1- *MdGDS-RG1*, AmpR | This study |
| pLJJ19 | Blunt, pGAL1- *LaGERDS*, AmpR | This study |
| pLJJ20 | Blunt, pGAL1- *TlTPS7414*, AmpR | This study |
| pLJJ21 | Blunt, pGAL1- *XsTPS1*, AmpR | This study |
| pLJJ22 | Blunt, pGAL1- *PnTPS3*, AmpR | This study |
| pLJJ23 | Blunt, pGAL1- *ZpTPS2*, AmpR | This study |
| pLJJ24 | Blunt, pGAL1- *STC1*, AmpR | This study |
| pLJJ25 | Blunt, pGAL1- *TcTPS8*, AmpR | This study |
| pLJJ26 | Blunt, pGAL1- *DcTPS7*, AmpR | This study |
| pLJJ27 | Blunt, pGAL1- *Actps1*, AmpR | This study |
| pLJJ28 | pCas, gRNA_*rox1*, AmpR | This study |
| pLJJ29 | pRS426, pGAL10- *Actps1*, pGAL1- ERG20, pGAL7-tHMG1, *URA*, AmpR | This study |
| pLJJ30 | pCas, gRNA_*exg1*, gRNA_*dpp1,* AmpR | This study |
| pLJJ31 | pCas, gRNA_*EGR9*, AmpR | This study |
| pLJJ32 | pRS426, pGAL1- *Actps1*, pGAL10- tHMG1, *URA*, AmpR | This study |
| pLJJ33 | pRS426, pGAL1- *Actps1*, pGAL10- tHMG1, *URA*, AmpR | This study |
| pLJJ34 | pRS426, pHXT1- *ERG9* | This study |

**Table S3** Primers used in this study.

| **Primer name** | **Primer sequence 5’-3’** |
| --- | --- |
| RT-Actps1-F | ATGACCCAGACGGTCACACGCCC |
| RT-Actps1-R | TCAACCTCTTACTAGAACAGTCTCCTCCTT |
| RT-Actps2-F | ATGTCCCAGGCTGTGCTTACAACCA |
| RT-Actps2-R | CTATAGAATAAACGTTTGGCTTCCATCCTC |
| RT-Actps3-F | ATGCCCACAGCAAAGCAAACCAAG |
| RT-Actps3-R | TTATAGCGTGACCACGACGCCGC |
| RT-Actps4-F | ATGTCTGTGAGCATGACAGTCTCGT |
| RT-Actps4-R | CTAGAGCGTGATCGATACACCGCCT |
| RT-Actps5-F | ATGGTATGAACCGTTTGAAAAAGCAAACA |
| RT-Actps5-R | TCAGGCTCTGCAAGTCTGCATCAGA |
| RTActin-F | AGTCCAAGCGTGGTATCC |
| RTActin-R | TAGAAGGCAGGGGCGTTG |
| pLJJ1-1-F | AAAAGCTGGAGCTCTAGTAGTTTAAACAGTCATGTAGCCGCCTAGCGAGCCTGGG |
| pLJJ1-1-R | GTTTTGGGACGCTCGAAGGCTTTAATTTGCGTAGTGCTGTCTGAACAGAATAAATG |
| pLJJ1-2-F | GCAAATTAAAGCCTTCGAGCGTCCCAAAAC |
| pLJJ1-2-R | ACAGGCCCCTTTTCCTTTGTCGATATCATGT |
| pLJJ1-3-F | ATGATATCGACAAAGGAAAAGGGGCCTGTTCAACCTCTGACCAAGACGGTTTCTTC |
| pLJJ1-3-R | AGTAAGAATTTTTGAAAATTCAATATAAATGACACAAACTGTTACCAGACCAGTTCC |
| pLJJ1-4-F | TATATTGAATTTTCAAAAATTCTTACTT |
| pLJJ1-4-R | TTGAGCGGTCCATTCGTCTATTAAGATTATAGTTTTTTCTCCTTGACGTTAAAGTAT |
| pLJJ1-5-F | TTTAACGTCAAGGAGAAAAAACTATAATCTTAATAGACGAATGGACCGCTCAAG |
| pLJJ1-5-R | CATTCAGGCTGCGCAACTGTTGTTTAAACGAGAAACTAGGCTAGTTTTAGCGGTG |
| pLJJ1-6-F | AGCCTAGTTTCTCGTTTAAACAACAGTTGCGCAGCCTGAATGGCGAATGGCGCGAC |
| pLJJ1-6-R | CCCAGGCTCGCTAGGCGGCTACATGACTGTTTAAACTACTAGAGCTCCAGCTTTTG |
| pLJJ2-3-F | ATATCGACAAAGGAAAAGGGGCCTGTTCATAAGATGAAAGTTTGAGAACCATC |
| pLJJ2-3-R | GTGGTGGTCAAAACAGCTTGGGACATTTATATTGAATTTTCAAAAATTCTT |
| pLJJ3-3-F | ATATCGACAAAGGAAAAGGGGCCTGTTCATAGAGTGACAACAACACCAC |
| pLJJ3-3-R | AAGAATTTTTGAAAATTCAATATAAATGCCAACCGCTAAGCAAACCAAGGCT |
| pLJJ4-3-F | ATGATATCGACAAAGGAAAAGGGGCCTGTCAAAGTAATAGAAACACCACCCTCC |
| pLJJ4-3-R | TAAGAATTTTTGAAAATTCAATATAAATGTCTGTCTCTATGACTGTCTCTTCT |
| pLJJ5-3-F | TGATATCGACAAAGGAAAAGGGGCCTGTCTCGAGAGCTCTACAAGTTTGCA |
| pLJJ5-3-R | TAAGAATTTTTGAAAATTCAATATAAATGGCTGCTGATCCAACTGTTACT |
| pLJJ6-F | CAGGCGCGCCGTGTACACGAGCTCTCAACCTCTGACCAAGACGGTTTCTTCCTTTTC |
| pLJJ6-R | AGAGTCCGGATCCCAATTGGGAGCTCATGACACAAACTGTTACCAGACCAGTT |
| pLJJ7-F | TATCGACAAAGGAAAAGGGGCCTGTTTAAATCTTGACAGACTCAATCAAA |
| pLJJ7-R | AAGAATTTTTGAAAATTCAATATAAATGGCTGCTTCTTTCGCTAATAAGT |
| pLJJ8-F | TATCGACAAAGGAAAAGGGGCCTGTTTACATTGGAACTGGGTCAATCAAC |
| pLJJ8-R | AAGAATTTTTGAAAATTCAATATAAATGTCTGTCGAGGGTTCTGCTATTT |
| pLJJ9-F | ATCGACAAAGGAAAAGGGGCCTGTTTAGACGGAAATGGCGTTGATGAAGC |
| pLJJ9-R | AAGAATTTTTGAAAATTCAATATAAATGGCTGCTAAACAAGGTGAAGTTG |
| pLJJ10-F | ATCGACAAAGGAAAAGGGGCCTGTTTATTTAATTGGATGGACCAAGACAC |
| pLJJ10-R | AAGAATTTTTGAAAATTCAATATAAATGACTAATATGTTTGCTTCTGCTG |
| pLJJ11-F | TCGACAAAGGAAAAGGGGCCTGTTTAGACAGACATGGCGTTAATGAAGC |
| pLJJ11-R | GCTTCGACATGTTTAGCAGCCATTTATATTGAATTTTCAAAAATTCTT |
| pLJJ12-F | TCGACAAAGGAAAAGGGGCCTGTTTAAATTGGGACTGGGTCAATCAGC |
| pLJJ12-R | TAAGAATTTTTGAAAATTCAATATAAATGTCTGTTCAGTCTTCTGGTGT |
| pLJJ13-F | TCGACAAAGGAAAAGGGGCCTGTTTAGGGAATTGGGTGGATGTACAAC |
| pLJJ13-R | TAAGAATTTTTGAAAATTCAATATAAATGGAGCTGAAAAATCAGTCTGTC |
| pLJJ14-F | ATATCGACAAAGGAAAAGGGGCCTGTTTAGTTAATTGGCATAGAGTCAA |
| pLJJ14-R | AAGAATTTTTGAAAATTCAATATAAATGCAATTGCCATGTGCTCAAGC |
| pLJJ15-F | GACAAAGGAAAAGGGGCCTGTTTAAATGACGGCGTTAATCAAGAC |
| pLJJ15-R | GTAAGAATTTTTGAAAATTCAATATAAATGTCTGGTCAAGTTTTGGCTTC |
| pLJJ16-F | TCGACAAAGGAAAAGGGGCCTGTTTAATGGCTGAAATTTGTGCTTCTGC |
| pLJJ16-R | AAGAATTTTTGAAAATTCAATATAAAATATGATACTCAATAGAGTGAATT |
| pLJJ17-F | ATCGACAAAGGAAAAGGGGCCTGTTTACAAAGCGACTGGCTCGACCAGA |
| pLJJ17-R | AAGAATTTTTGAAAATTCAATATAAATGTCTTCTGTCGAAGTTCCATTGT |
| pLJJ18-F | ATCGACAAAGGAAAAGGGGCCTGTTTAAATAGTCAATGGCTCGACGAAA |
| pLJJ18-R | GTAAGAATTTTTGAAAATTCAATATAAATGGAGATTCATTCTTCTGTTGT |
| pLJJ19-F | ATCGACAAAGGAAAAGGGGCCTGTTTAGACTGGGACGGGGTCCAACAAA |
| pLJJ19-R | AAGAATTTTTGAAAATTCAATATAAATGGCTATGTGTGTCAATTCTACT |
| pLJJ20-F | ATCGACAAAGGAAAAGGGGCCTGTTTAAATGGACATGGAGATAATGAA |
| pLJJ20-R | AGAATTTTTGAAAATTCAATATAAATGGAAGTTAAGCAAGAGGTTTTAA |
| pLJJ21-F | ATCGACAAAGGAAAAGGGGCCTGTTTACAATGGAATTGGTTGAACCAAA |
| pLJJ21-R | AAGAATTTTTGAAAATTCAATATAAATGGGTTTTTCTTTCGTCACTAA |
| pLJJ22-F | ATCGACAAAGGAAAAGGGGCCTGTTTACAATGGAATTGGTTGAACCAAA |
| pLJJ22-R | GAATTTTTGAAAATTCAATATAAATGGGTTTTTCTTTCGTCACTAATG |
| pLJJ23-F | ATCGACAAAGGAAAAGGGGCCTGTTTACAATGGAACTGGGTTAATCAAC |
| pLJJ23-R | AGAATTTTTGAAAATTCAATATAAATGTCTTGTAATGTCTCTGCTGCTC |
| pLJJ24-F | TCGACAAAGGAAAAGGGGCCTGTTTAACCCTTCAAAATGGTGAAGTAGA |
| pLJJ24-R | AAGAATTTTTGAAAATTCAATATAAATGGCTTACAAACCAGGTAGAGA |
| pLJJ25-F | ATCGACAAAGGAAAAGGGGCCTGTTTAAATTTGAATTGGGTCAATCAA |
| pLJJ25-R | GTCGTCACCTTTCAAAGAAGTCATTTATATTGAATTTTCAAAAATTCT |
| pLJJ26-F | TCGACAAAGGAAAAGGGGCCTGTTTAAATTGGGAAGGGGTCCAACAA |
| pLJJ26-R | AGAATTTTTGAAAATTCAATATAAATGTATGTCAATTCTACTTCTGGT |
| pLJJ28-1-F | \| GGTCTCGGATCTGTTCAGACAGCACTACCACGTTTTAGAGCTAGAAATAGCA \| \| --- \| |
| pLJJ28-1-R | \| GGTCTCTTGCGCAAGCCCGGAATCGAACC \| \| --- \| |
| pLJJ28-2-F | \| GGTCTCACGCATTTTTTTGGATCCATCTAAAGTCATTTCA \| \| --- \| |
| pLJJ28-2-R | GGTCTCAAAACCTTTTTCGATGATGTAGTTTCTGG |
| pLJJ29-4-R | TCTCTCCTAATTTCTTTTTCTGAAGCCATTATAGTTTTTTCTCCTTGACGTT |
| pLJJ29-5-F | AACGTCAAGGAGAAAAAACTATAATGGCTTCAGAAAAAGAAATTAGGAGAGA |
| pLJJ29-5-R | CTATTTGCTTCTCTTGTAAACTTTGTTCAAG |
| pLJJ29-6-F | GAACAAAGTTTACAAGAGAAGCAAATAGTTTGCCAGCTTACTATCCTTCTTG |
| pLJJ29-6-R | GAAATGACTGTTTTATTGGTTAAAACCATTTTTGAGGGAATATTCAACTG |
| pLJJ29-7-F | ATGGTTTTAACCAATAAAACAGTCATTTCTGG |
| pLJJ29-7-R | TTAGGATTTAATGCAGGTGACGGACCC |
| pLJJ29-8-F | GGGTCCGTCACCTGCATTAAATCCTAAGCGAATTTCTTATGATTTATGATT |
| pLJJ29-8-R | CCGGTAGAGGTGTGGTCAATAAGAGC |
| pLJJ29-9-F | GCTCTTATTGACCACACCTCTACCGGATCTTAATAGACGAATGGACCGCTCAAGGTGT |
| pLJJ29-9-R | CGCCATTCGCCATTCAGGCTGCGCAACTGTTGTTTAAACGAGAAACTAGG |
| pLJJ30-1-F | GGTCTCACGCACCAATCCGTGGTGTCAACATGTTTTAGAGCTAGAAATAGCA |
| pLJJ30-1-R | GGTCTCTATCCAAAAAAATGCGCAAGCCCGGAATCGAACCG |
| pLJJ30-2-F | GGTATCAGGATCCATCTAAAGTCATTTCAAC |
| pLJJ30-2-R | GGTCTCTGAGGGATCATTTATCTTTCACTGCGGAGAA |
| pLJJ30-3-F | GGTCTCACCTCATCCGGGATCCTCTGAGTTTTAGAGCTAGAAATAGCAAGTTAAA |
| pLJJ30-3-R | GGTCTCAAAACAAAAAAATGCGCAAGCCCGGAATCGAACCGG |
| pLJJ31-1-F | GGTCTCGGATCGAGCAGCGAGAACACGACCACGGGTTTTAGAGCTAGAAATAGCAAG |
| pLJJ31-1-R | GGTCTCTTGCGCAAGCCCGGAATCGAACC |
| pLJJ31-2-F | GGTCTCACGCATTTTTTTGGATCCATCTAAAGTCATTTCA |
| pLJJ31-2-R | GGTCTCAAAACCTTTTTCGATGATGTAGTTTCTGG |
| pLJJ32-1-F | CAAAAGCTGGAGCTCTAGTAGTTTAAACTAATAGTACGTAATGTAGGGAGCCTGCTTC |
| pLJJ32-1-R | TTTGGGACGCTCGAAGGCTTTAATTTGCTTGACACCACGGATTGGTTCTCCGAGGGAA |
| pLJJ32-2-F | GCAAATTAAAGCCTTCGAGCGTCCCAAA |
| pLJJ32-2-R | ACAGGCCCCTTTTCCTTTGTCGAT |
| pLJJ32-3-F | ATCGACAAAGGAAAAGGGGCCTGTTTAGGATTTAATGCAGGTGACGGACCCA |
| pLJJ32-3-R | ATGGTTTTAACCAATAAAACAGTCA |
| pLJJ32-4-F | TGACTGTTTTATTGGTTAAAACCATTTATATTGAATTTTCAAAAATTCTTAC |
| pLJJ32-4-R | TATAGTTTTTTCTCCTTGACGTTAAAGT |
| pLJJ32-5-F | ACTTTAACGTCAAGGAGAAAAAACTATAATGACACAAACTGTTACCAGACCA |
| pLJJ32-5-R | CGATTTCAATTCAATTCAATTCAACCTCTGACCAAGACGGTTTCTTCCTTT |
| pLJJ32-6-F | ATTGAATTGAATTGAAATCGATAGAT |
| pLJJ32-6-R | AACGAACGCAGAATTTTCGAGTTATTAAA |
| pLJJ32-7-F | TTTAATAACTCGAAAATTCTGCGTTCGTTTGGTTGGTTACTTCTTGAACCA |
| pLJJ32-7-R | CCATTCGCCATTCAGGCTGCGCAACTGTTGTTTAAACTTGTAACACCAGATAATCCAA |
| pLJJ32-8-F | TGGATTATCTGGTGTTACAAGTTTAAACAACAGTTGCGCAGCCTGAATGGCGAATGGC |
| pLJJ32-8-R | GAAGCAGGCTCCCTACATTACGTACTATTAGTTTAAACTACTAGAGCTCCAGCTTTTG |
| pLJJ33-1-F | TTTTATTGTTTCCTGTTGTTTTTCTCTTTC |
| pLJJ33-1-R | TGGGACGCTCGAAGGCTTTAATTTGCGAGGATCCCGGATGAGGAATTACATCCT |
| pLJJ33-2-F | GCAAATTAAAGCCTTCGAGCGTCCCAAA |
| pLJJ33-7-R | AACGAACGCAGAATTTTCGAGTTATTAAAC |
| pLJJ33-8-F | TTTAATAACTCGAAAATTCTGCGTTCGTTGTGACTGCTTCCTCCAGGGTGACATCTGA |
| pLJJ33-8-R | CCATTCGCCATTCAGGCTGCGCAACTGTTGTTTAAACTTTGTCGATCGGTTGTATATT |
| pLJJ33-9-F | AATATACAACCGATCGACAAAGTTTAAACAACAGTTGCGCAGCCTGAATGGCGAATGG |
| pLJJ33-9-R | AAAGAGAAAAACAACAGGAAACAATAAAAGTTTAAACTACTAGAGCTCCAGCTT |
| pLJJ34-1-F | GTTTAAACGTTCCGATTGCAGTTGGAATGCAAATG |
| pLJJ34-1-R | CAGGAGGGGGGAATTATATAAAAGAAAAAGGGCAAAGCAAATAGGATGGTAAG |
| pLJJ34-2-F | GCTTTGCCCTTTTTCTTTTATATAATTCCCCCCTCCTGAAGCAAA |
| pLJJ34-2-R | GGATGCAATGCCAATTGTAATAGCTTTCCCATGATTTTACGTATATCAACTAGTTGAC |
| pLJJ34-3-F | ATGGGAAAGCTATTACAATTGGCATTGCATCC |
| pLJJ34-3-R | GTTTAAACTTGATTTCAAAATTAAATAGCAGGTAGTAC |

**Table S4** Terpene synthases used for phylogenetic analysis.

| Organism | Accession number |
| --- | --- |
| *Arabidopsis thaliana* | AT2G24210 |
|  | AT4G16740 |
|  | AT4G16730 |
|  | AT1G61680 |
|  | AT1G79460 |
|  | AT1G61120 |
|  | AT3G25820 |
| *populus trichocarpa* | Pt0004s02970 |
|  | Pt0004s03810 |
|  | Pt0002s05300 |
|  | Pt0005s23190 |
|  | Pt0008s08190 |
| *Selaginella moellendorffii* | Selmol_112927 |
|  | Selmol_402351 |
|  | Selmol_407280 |
|  | Selmol_412139 |
|  | Selmol_418910 |
| *Ginkgo biloba* | AAL09965 |
| *Abies grandis* | AAF61453 |
|  | AAF61454 |
|  | AAB71085 |
|  | AAB70707 |
|  | AAC05727 |
|  | AAC05728 |
|  | AAC24192 |
|  | AAB05407 |

**Table S5** The specific rotation of the product of AcTPS1 (G) and (-)-Germacrene D standard (S).

| NO. | Name | C (mg/mL) | L (dm) | [α] | solvent | Temp |
| --- | --- | --- | --- | --- | --- | --- |
| 1 | G | 0.25 | 1 | -28 | CHCl_3_ | 20.00 |
| 2 | G | 0.25 | 1 | -32 | CHCl_3_ | 20.00 |
| 3 | G | 0.25 | 1 | -32 | CHCl_3_ | 20.00 |
| 4 | S | 0.26 | 1 | -42.307 | CHCl_3_ | 20.00 |
| 5 | S | 0.26 | 1 | -46.153 | CHCl_3_ | 20.00 |
| 6 | S | 0.26 | 1 | -46.153 | CHCl_3_ | 20.00 |

**Table S6** The germacrene D production in different engineered yeast strains

| Strain | Production (mg/L) | Protein | Organism |
| --- | --- | --- | --- |
| SC1 | 2.3±0.1 | SSLH2 | *Lycopersicon esculentum* |
| SC2 | 7.4±0.1 | PtdTPS1 | *Populus trichocarpa X Populus deltoides* |
| SC3 | 134.3±14.1 | Sc19 | *Solidago canadensis* |
| SC5 | 2.5±0.4 | Sc11 | *Solidago canadensis* |
| SC9 | 2.3±0.1 | VvGwGerD | *Vitis vinifera* |
| SC12 | 2.3±0.3 | MdGDS-RG1 | *Malus domestica* |
| SC13 | 11.4±0.1 | LaGERDS | *Lavandula angustifolia* |
| SC15 | 40.3±4.6 | XsTPS1 | *Xanthium strumarium* |
| SC18 | 2.2±0.1 | STC1 | *Fusarium fujikuroi* |
| SC20 | 7.6±0.1 | DcTPS7 | *Daucus carota L.* |
| SC21 | 344.4±32.6 | AcTPS1 | *Acremonium chrysogenum* |

**
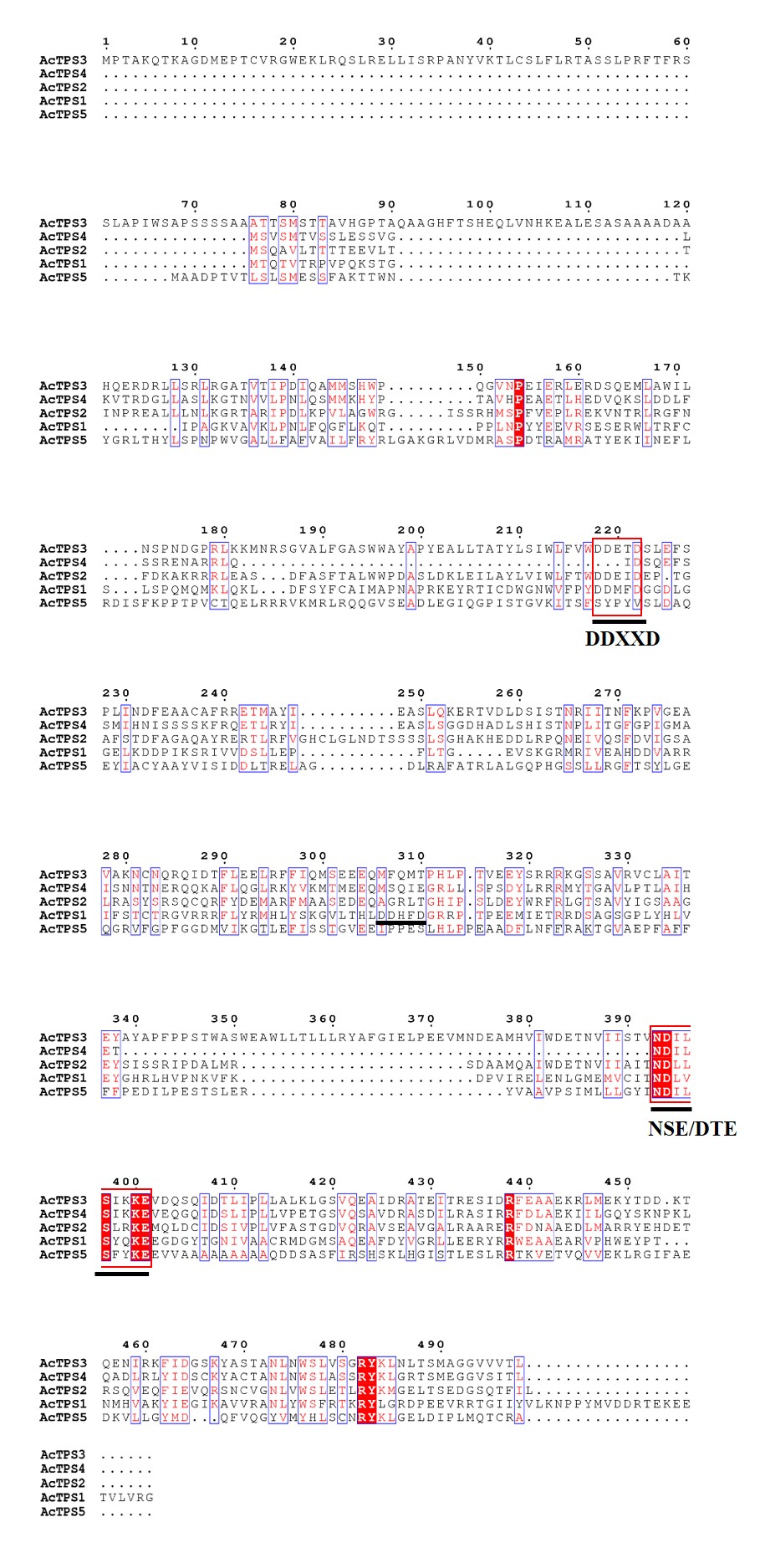
**

**Fig. S1** Amino acids alignment of the sesquiterpene synthases from *Acremonium chrysogenum*. The conserved motifs are marked respectively.


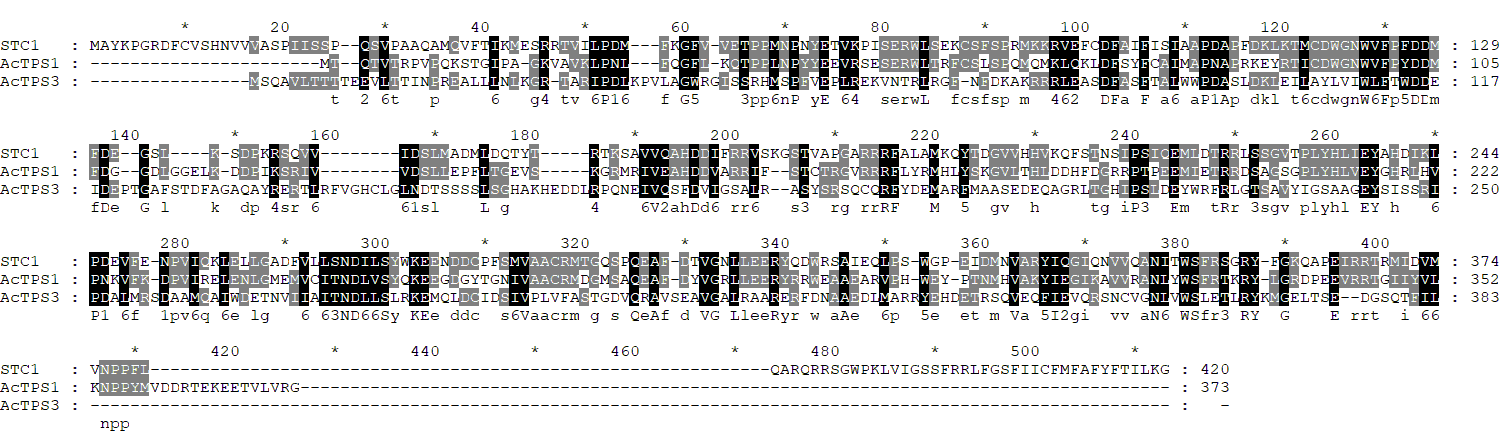


**Fig. S2** Amino acids alignment of STC1, AcTPS1 and AcTPS3.


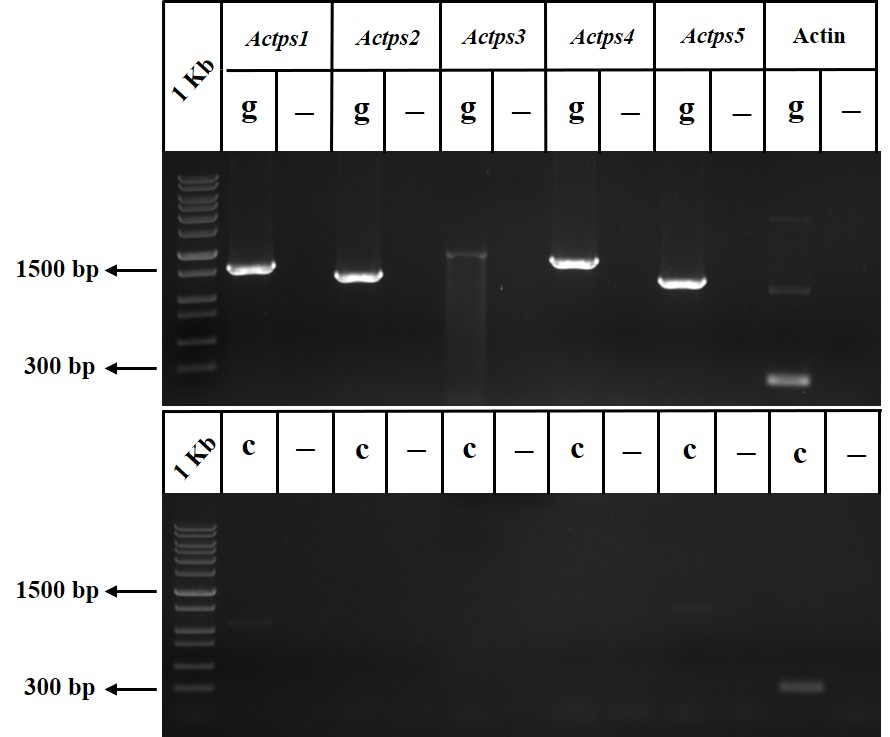


**Fig. S3** The transcription of *Actps1* to *Actps5* in *Acremonium chrysogenum*. g, genome DNA; c, cDNA; -, negative control.


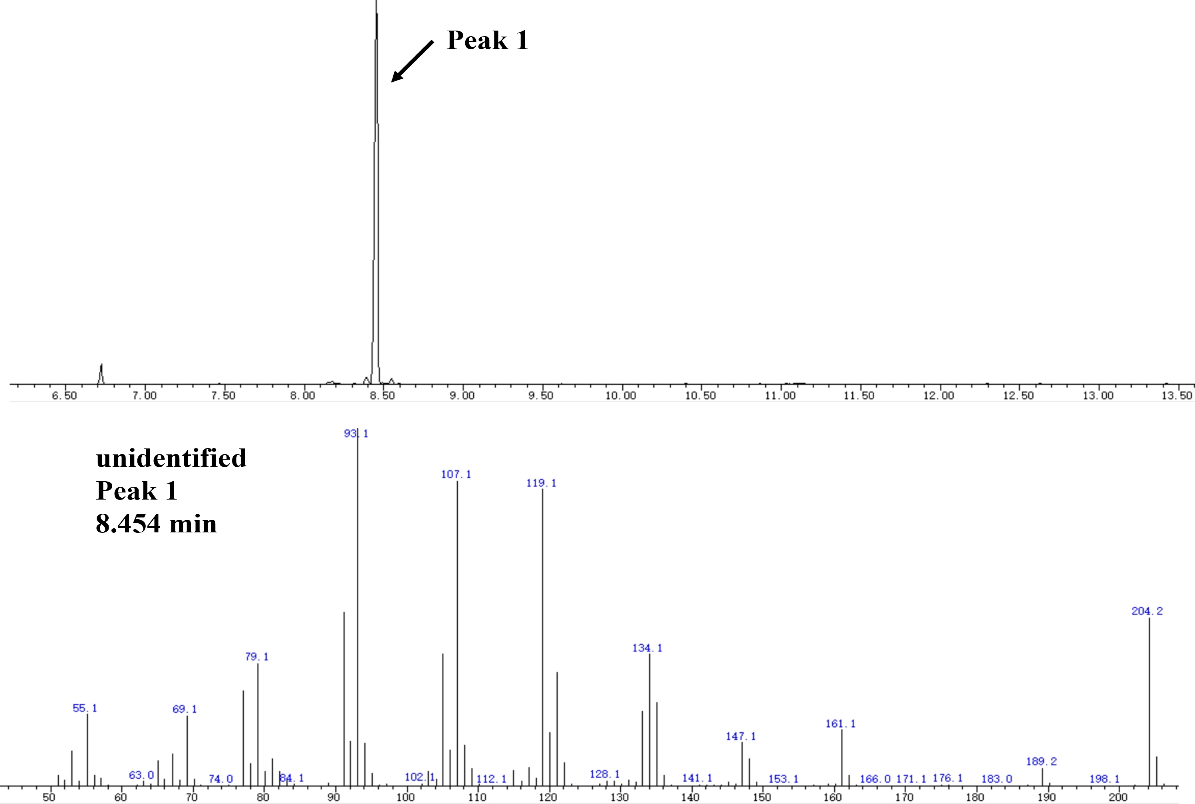


**Fig. S4** GC spectrum and the corresponding Mass spectra of sesquiterpenes biosynthesized by AcTPS5. The retention time of the main peak1 is shown with the corresponding Mass spectra.


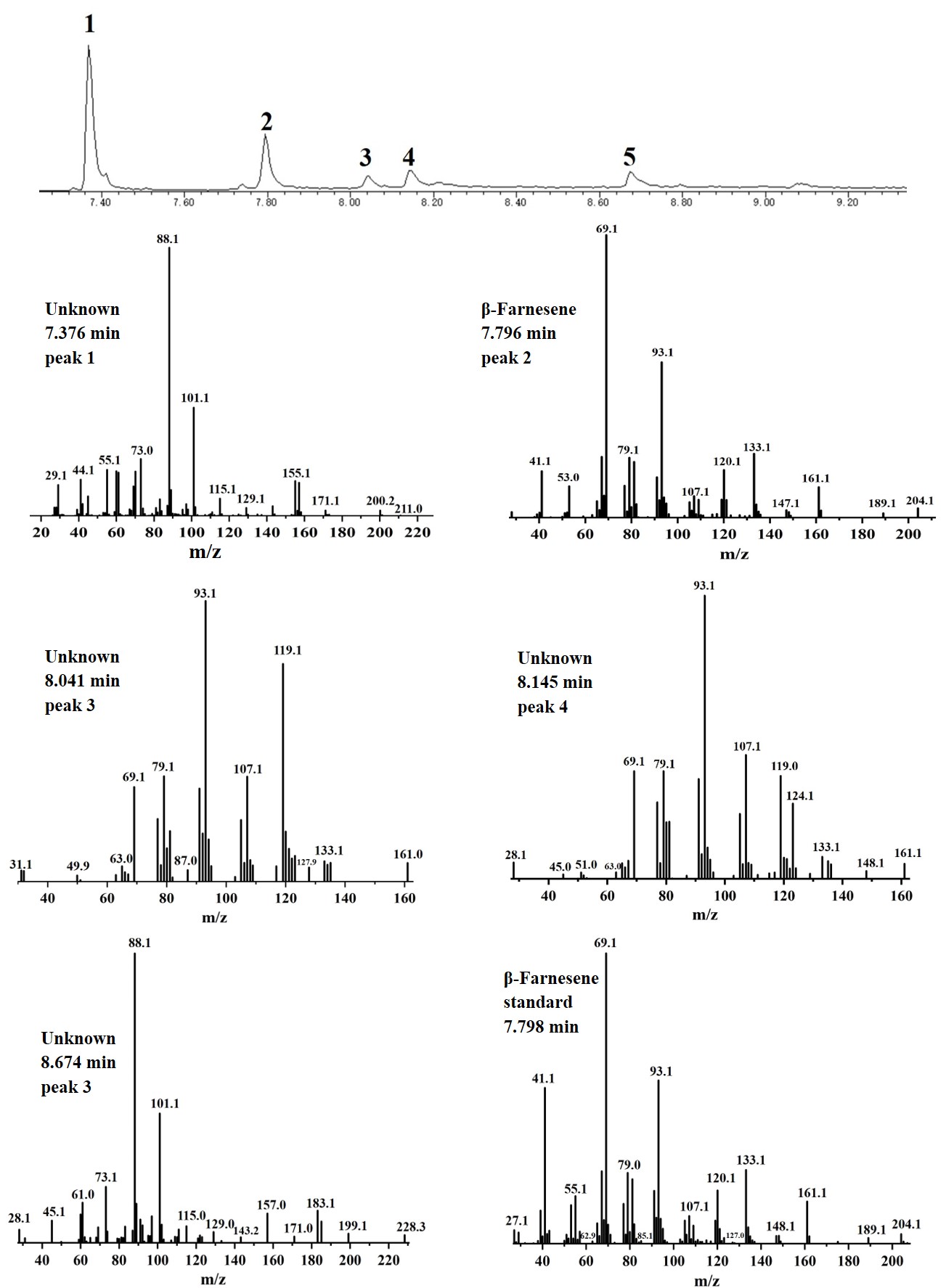


**Fig. S5** GC spectrum and the corresponding Mass spectra of sesquiterpenes biosynthesized by AcTPS2. Peak 1 to peak 5 represent multiple products synthesized. their retention times are shown with the corresponding Mass spectra. Compounds were identified by comparison with the NIST database (National Institute of Standards and Technology) library and β-farnesene standard.


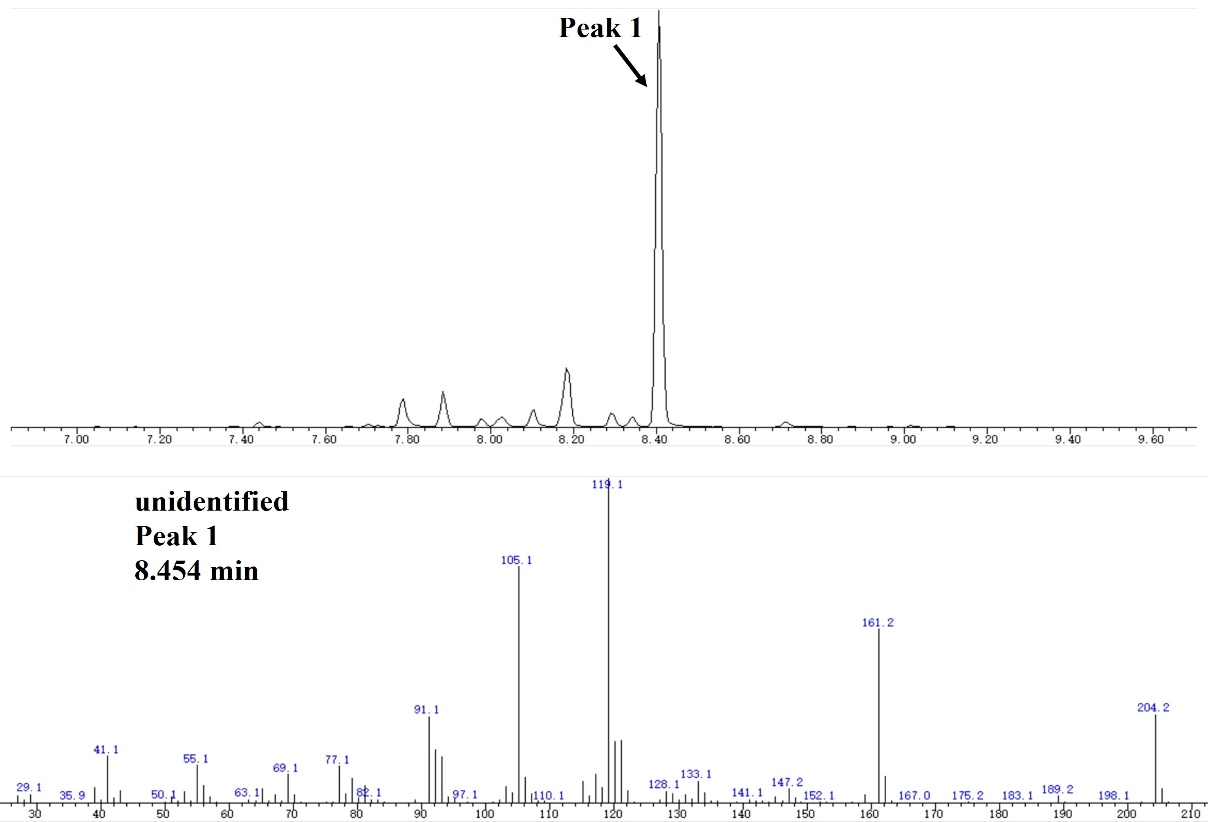


**Fig. S6** GC spectrum and the corresponding Mass spectra of sesquiterpenes biosynthesized by AcTPS3. The retention time of the main peak1 is shown with the corresponding Mass spectra.


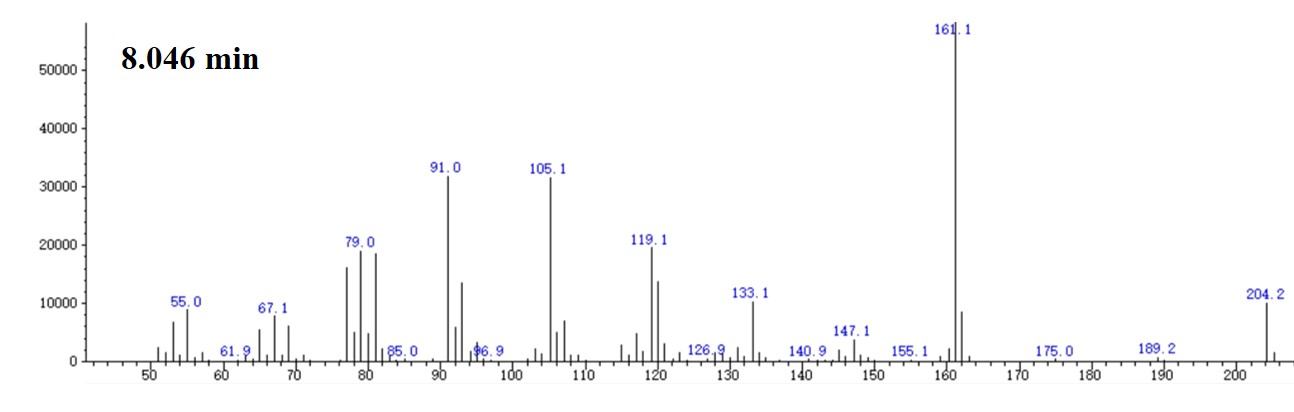


**Fig. S7** (-)-Germacrene D standard mass spectrum detected by GC-MS. shown by the 204 m/z ion in the chromatographic trace.


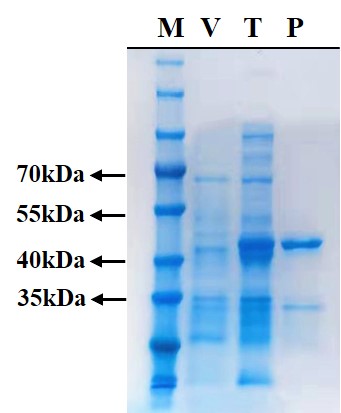


**Fig. S8** Purified AcTPS1 protein from recombinant *Escherichia coli*. M refers to the protein marker, V refers to the pET-45b total cell disruption components, T refers to the AcTPS1 total cell disruption components, P refers to the purified AcTPS1 protein.


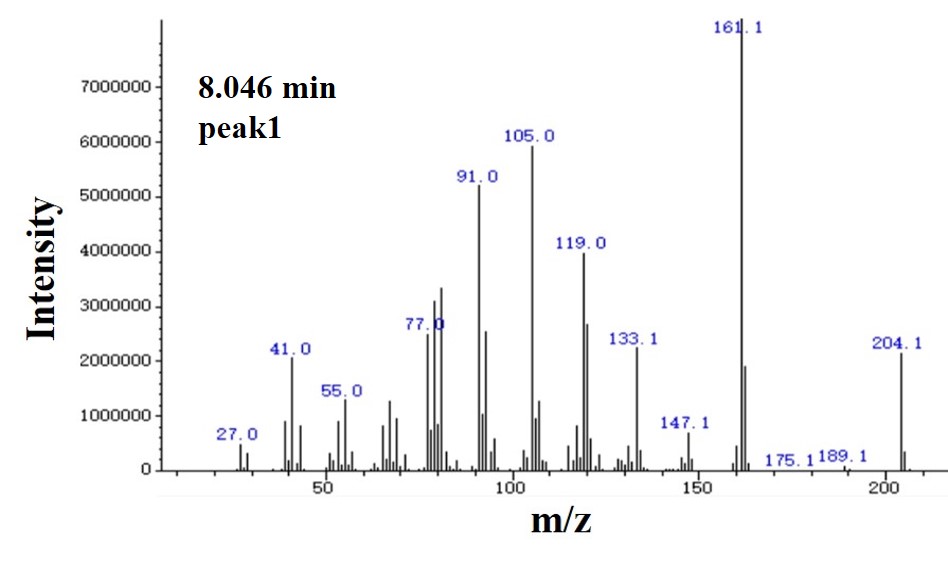


**Fig. S9** The mass spectra of the AcTPS1 enzymatic product.


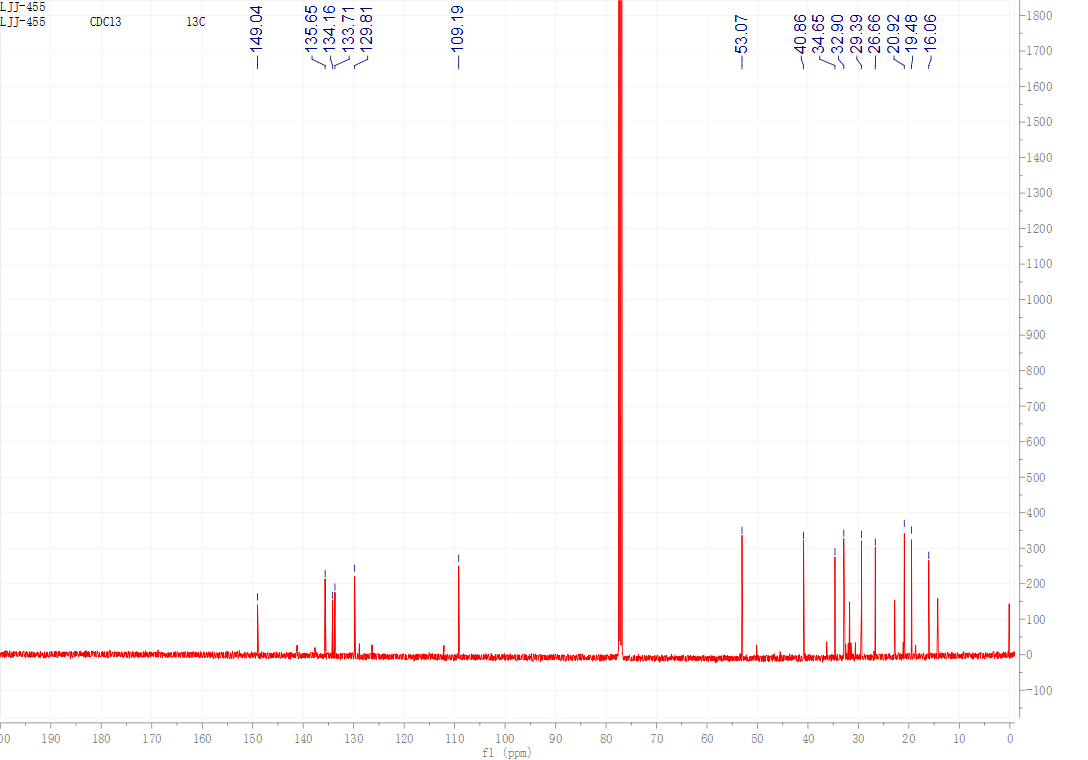


**Fig. S10** ^13^C NMR spectra of (-)-Germacrene D (400 MHz, CDCl_3_).


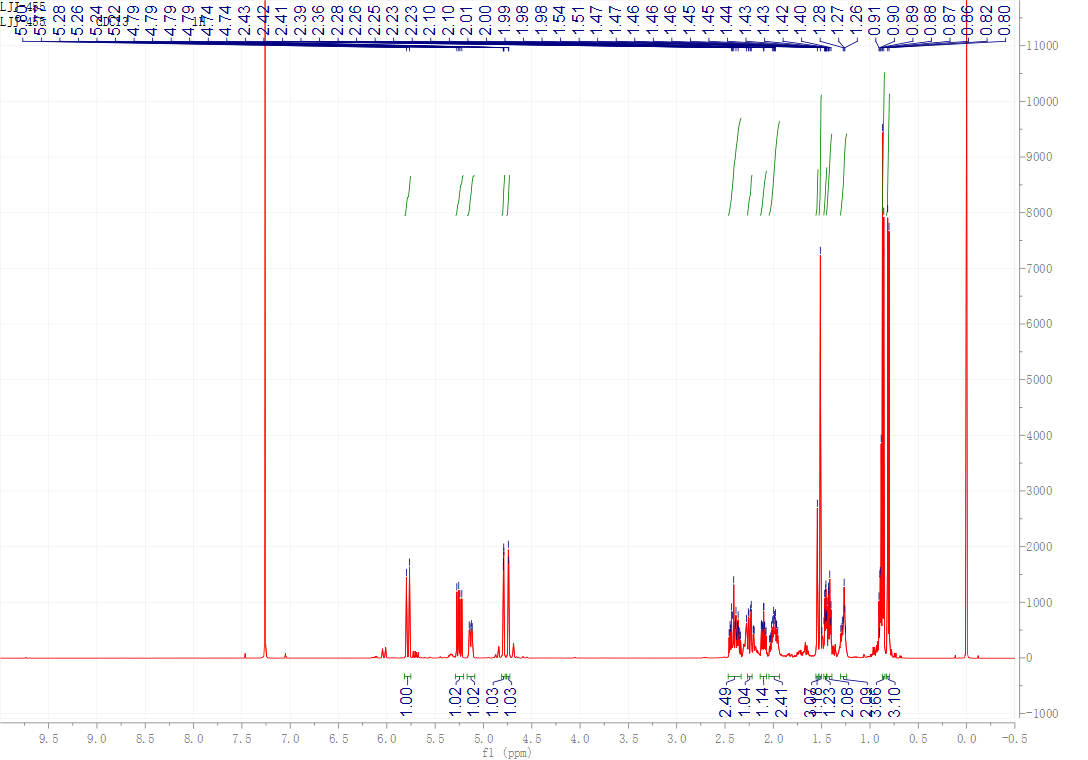


**Fig. S11** ^1^H NMR spectrum of (-)-Germacrene D (400 MHz, CDCl_3_).


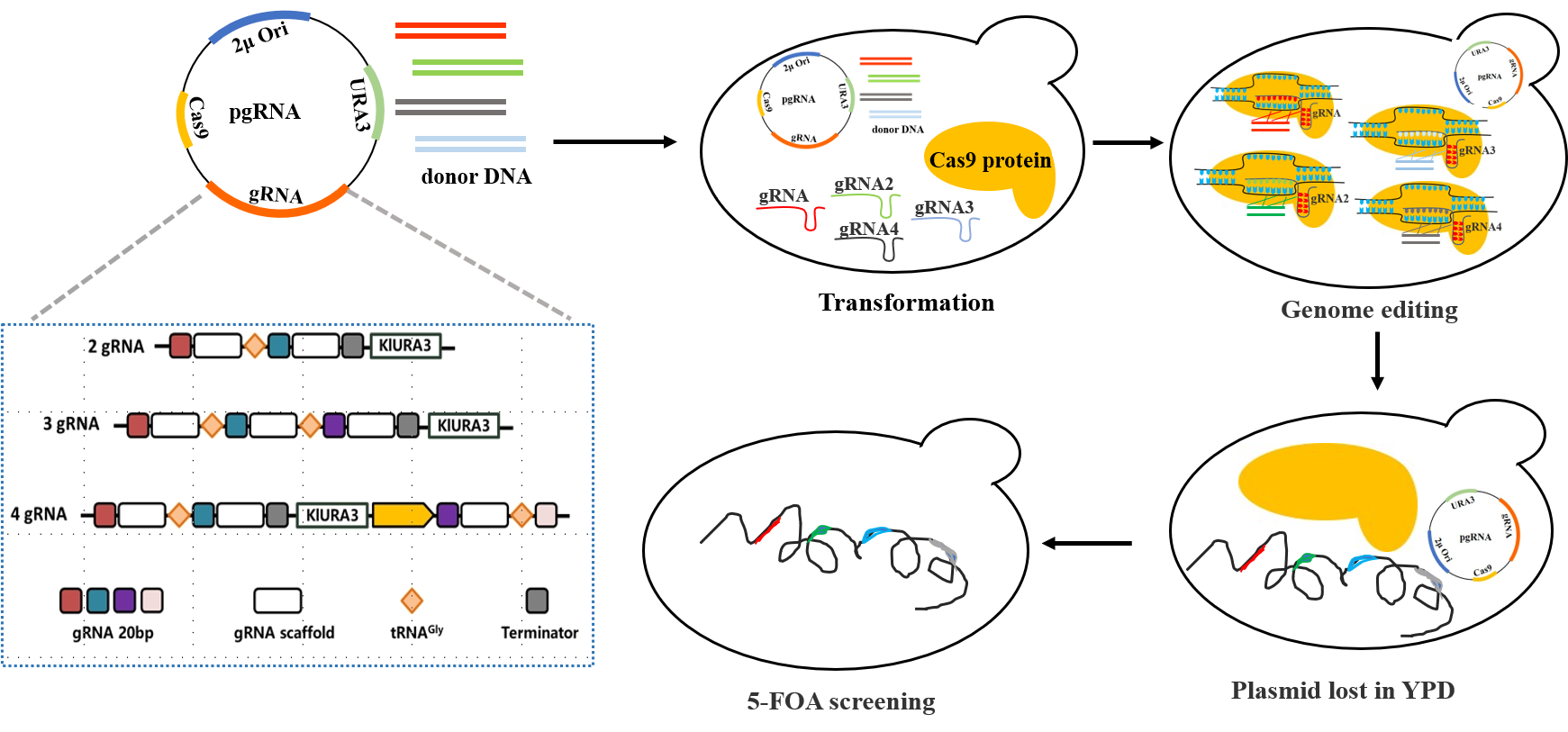


**Fig. S12** The schematic of gene editing with CRISPR/Cas9 system mediated by a recyclable gRNA plasmid. The gRNA plasmid pgRNA with URA3 gene as the selective marker and the donor DNA were transformed together into *S. cerevisiae* for genome editing. Then the gRNA plasmid pgRNA was lost by cultured in YPD medium combining screened with 5-FOA to fulfill recyclable utilization.


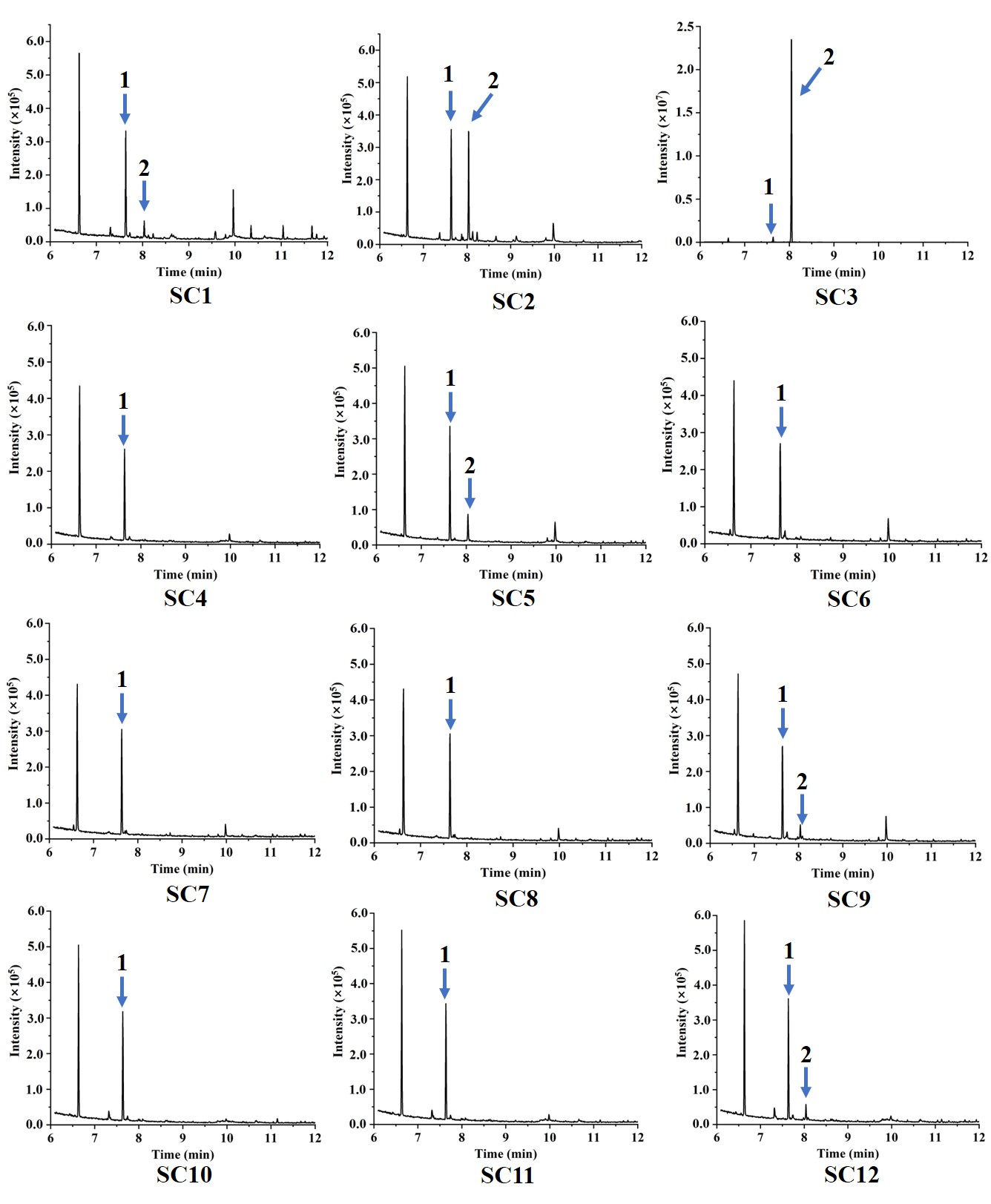


**Fig. S13** GC spectrum of Sc1-Sc12. Peak1, the internal standard β-caryophyllene (CAS# 87-44-5); Peak 2, germacrene D.


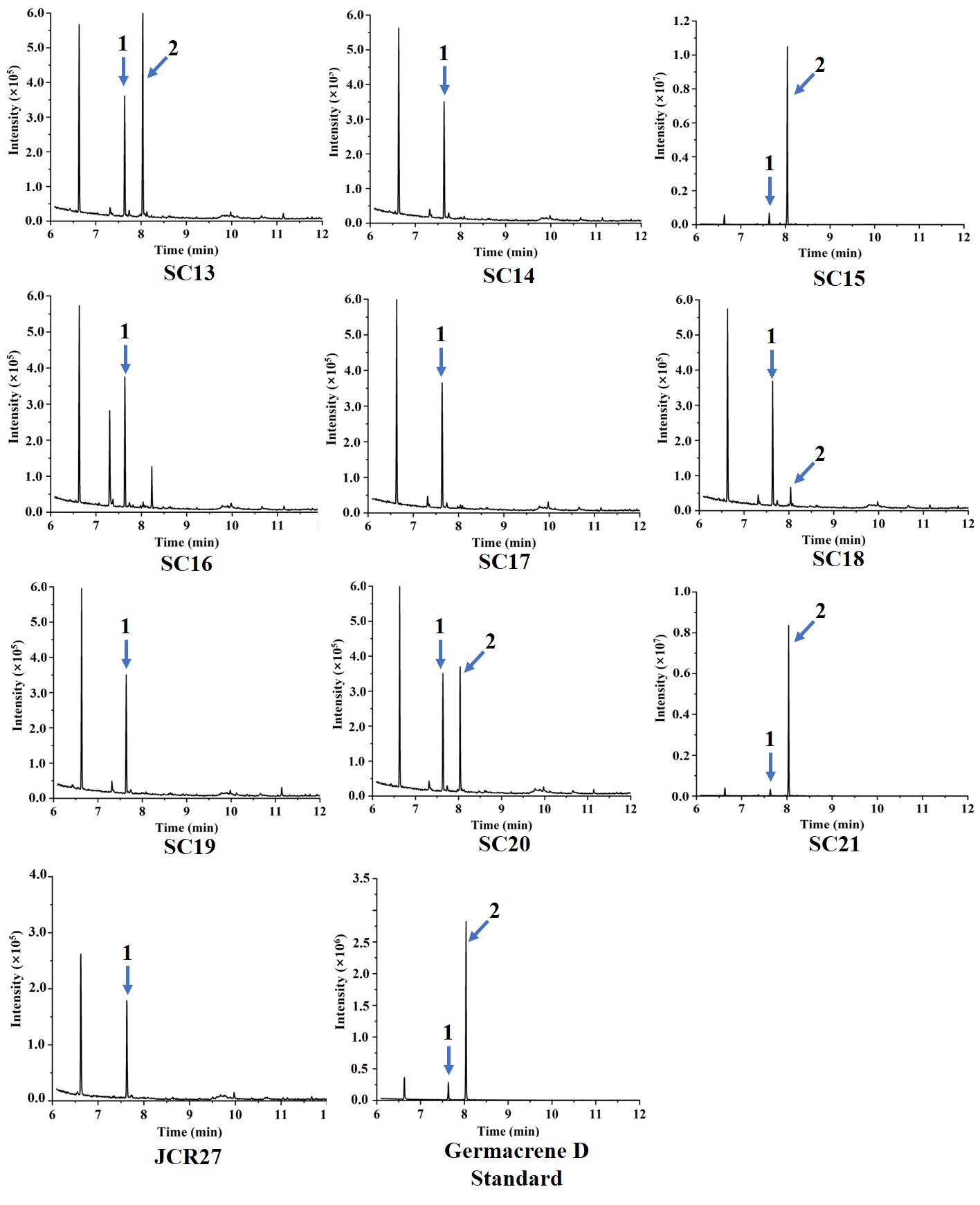


**Fig. S14** GC spectrum of Sc13-Sc21, JCR27 and germacrene D standard. The organic phase of SC21 cultures was diluted tenfold for quantitative analysis. Peak1, the internal standard β-caryophyllene (CAS# 87-44-5); Peak 2, germacrene D. JCR27, the start stain used in this study; Germacrene D standard, CAS# 37839-63-7.

**Supplementary references**

[1] Rutger S. van der H, Antonio JM, David B, Steven DT, John CS. Genetic control and evolution of sesquiterpene biosynthesis in *Lycopersicon esculentum* and *L. hirsutum*. Plant Cell. 2000; 12:2283-2294.

[2] Inna G, Moshe S, Naama M, Dan P, Mery DY, Gil S, Einat B, Olga D, Mariana O, Michal E, Wang JH, Zach A, Eran P, Efraim L, Dani Z, Alexander V, David W. Rose scent: genomics approach to discovering novel floral fragrance-related genes. Plant Cell. 2002; 14:2325-38.

[3] He XF, David E. Mechanism and stereochemistry of the germacradienol/germacrene D synthase of *Streptomyces coelicolor* A3(2). J Am Chem Soc. 2004; 126:2678-9.

[4] Gen-Ichiro A, Dezene H, Jörg B. Forest tent caterpillars (*Malacosoma disstria*) induce local and systemic diurnal emissions of terpenoid volatiles in hybrid poplar (*Populus trichocarpa* x *deltoides*): cDNA cloning, functional characterization, and patterns of gene expression of (-)-germacrene D synthase, PtdTPS1. Plant J. 2004; 37:603-16.

[5] Ian P, Iris GA, Andy LP, Wilfried AK, Harro JB, Michael HB. Enantiospecific (+)- and (-)-germacrene D synthases, cloned from goldenrod, reveal a functionally active variant of the universal isoprenoid-biosynthesis aspartate-rich motif. Arch Biochem Biophys. 2004; 432:136-44.

[6] Yoko I, Rachel DR, Eyal F, David RG, Einat B, Efraim L, Eran P. The biochemical and molecular basis for the divergent patterns in the biosynthesis of terpenes and phenylpropenes in the peltate glands of three cultivars of basil. Plant Physiol. 2004; 136: 3724-3736.

[7] Joost L, Pat B, Jörg B. Vitis vinifera terpenoid cyclases: functional identification of two sesquiterpene synthase cDNAs encoding (+)-valencene synthase and (-)-germacrene D synthase and expression of mono- and sesquiterpene synthases in grapevine flowers and berries. Phytochemistry. 2004; 65:2649-59.

[8] Fabienne D, Laurence P, Wu SQ, Anthony C, Joseph C, Michel S. The diverse sesquiterpene profile of patchouli, *Pogostemon cablin*, is correlated with a limited number of sesquiterpene synthases. Arch Biochem Biophys. 2006; 454:123-36.

[9] Picaud S, Olsson ME, Brodelius M, Brodelius PE. Cloning, expression, purification and characterization of recombinant (+)-germacrene D synthase from *Zingiber officinale*. Arch Biochem Biophys. 2006; 452:17-28.

[10] Niels JN, Mindy YW, Adam JM, Sol AG, Chen XY, Yar-Khing Y, Lesley LB, Dinesh AN, Natalia D, Ross GA. Two terpene synthases are responsible for the major sesquiterpenes emitted from the flowers of kiwifruit (*Actinidia deliciosa*). J Exp Bot. 2009; 60:3203-19.

[11] Diane MM, Sébastien A, Marina BS, Laurent D, Michel S, Omid T, Steven TL, Jörg B. Functional annotation, genome organization and phylogeny of the grapevine (*Vitis vinifera*) terpene synthase gene family based on genome assembly, FLcDNA cloning, and enzyme assays. BMC Plant Biol. 2010; 21:10-226.

[12] Christoph C, Julia A, Johannes N, Jonathan G, Jörg D. Terpene synthases of oregano (*Origanum vulgare L*.) and their roles in the pathway and regulation of terpene biosynthesis. Plant Mol Biol. 2010; 73:587-603.

[13] Holger D, G Andreas B, Sandra Ir, Joshua SY, Chen F, Jonathan G, Sybille BU, Tobias GK. Four terpene synthases produce major compounds of the gypsy moth feeding-induced volatile blend of *Populus trichocarpa*. Phytochemistry. 2011; 72:897-908.

[14] Adelene LSong, Janna OA, Mohd PA, Norazizah S, Raha AR. Functional expression of an orchid fragrance gene in *Lactococcus lactis*. Int J Mol Sci. 2012; 13:1582-97.

[15] Bryan WP, Hue TT, Benjamin P, Tegan MH, Gao ZZ, Gillian M, John CV, Soo-Un K, Dae-Kyun R. Enzymatic synthesis of valerena-4,7(11)-diene by a unique sesquiterpene synthase from the valerian plant (*Valeriana officinalis*). FEBS J. 2012; 279:3136-46.

[16] Sandra I, Sandra TK, Grit K, Jonathan G, Jörg D, Tobias GK. The organ-specific expression of terpene synthase genes contributes to the terpene hydrocarbon composition of chamomile essential oils. BMC Plant Biol. 2012; 12: 84.

[17] Niels JN, Sol AG, Chen XY, Estelle JB, Adam JM, Mindy YW, Ross GA. Functional genomics reveals that a compact terpene synthase gene family can account for terpene volatile production in apple. Plant Physiol. 2013; 161:787-804.

[18] Chen H, Li GL, Tobias GK, Qidong J, Jonathan G, Feng C. Positive Darwinian selection is a driving force for the diversification of terpenoid biosynthesis in the genus *Oryza*. BMC Plant Biol. 2014;14: 239.

[19] Frédéric J, Sandrine M, Aurélie B, Sylvain L, Cécile P, Tarek B, Kévin P, Sébastien F, Yann G, Florence N, Sylvie B, Jean-Louis M. Isolation and functional characterization of a τ-cadinol synthase, a new sesquiterpene synthase from *Lavandula angustifolia*. Plant Mol Biol. 2014; 84:227-41.

[20] A Trine，C Federico，S Henrik. Optimization of biochemical screening methods for volatile and unstable sesquiterpenoids using HS-SPME-GC-MS. Chromatography. 2015; 2:277-292.

[21] Li YJ, Chen FF, Li ZQ, Li CF, Zhang YS. Identification and functional characterization of sesquiterpene synthases from *Xanthium strumarium*. Plant Cell Physiol. 2016; 57:630-41.

[22] Jin ZH, Moonhyuk K, Ah-Reum L, Dae-Kyun R, Juraithip W, Soo-Un K. Molecular cloning and functional characterization of three terpene synthases from unripe fruit of black pepper (*Piper nigrum*). Arch Biochem Biophys. 2018. 15; 638:35-40.

[23] Fujita Y, Koeduka T, Aida M, Suzuki H, Iijima Y, Matsui K. Biosynthesis of volatile terpenes that accumulate in the secretory cavities of young leaves of Japanese pepper (*Zanthoxylum piperitum*): Isolation and functional characterization of monoterpene and sesquiterpene synthase genes. Plant Biotechnol. 2017; 34:17-28.

[24] Niehaus EM, Schumacher J, Burkhardt I, Rabe P, Spitzer E, Münsterkötter M, Güldener U, Sieber CM, Dickschat JS, Tudzynski B. The GATA-type transcription factor *Csm1* regulates conidiation and secondary metabolism in *Fusarium fujikuroi.* Front Microbiol. 2017; 8:1175.

[25] Bjørn D, Damian PD, Crystal S, Henrik TS. In planta and in silico characterization of five sesquiterpene synthases from *Vitis vinifera* (cv. Shiraz) berries. Planta. 2019; 249:59-70.

[26] Niu Y, Yang JC, Yin GT, Li RS, Zou WT. Transcriptome analysis of oleoresin-producing tree *Sindora Glabra* and characterization of sesquiterpene synthases. Front Plant Sci. 2018; 9:1619.

[27] Jun IH, Kazutoshi S, Tetsuya S, Norihiko M. Isolation and functional characterization of new terpene synthase genes from traditional edible plants. J Oleo Sci. 2018; 67:1235-1246.

[28] Ma LT, Yi RL, Liu PL, Cheng YT, Tz-Fan S, Nai WT, Wang SY, Chu FH. Phylogenetically distant group of terpene synthases participates in cadinene and cedrane-type sesquiterpenes accumulation in *Taiwania cryptomerioides*. Plant Sci. 2019; 289: 110277.

[29] Andrew M, Chen X, John TL, Tobias GK, Kyle AP, Philipp Z, Meredith R, LeMar C, Suzanne L, Chen F, Dorothea T. Biosynthesis and emission of stress-induced volatile terpenes in roots and leaves of switchgrass ( *Panicum virgatum L.*). Front Plant Sci. 2019; 10: 1144.

[30] Ling CC, Zheng LJ, Yu XR, Wang HH, Wang CX, Wu HY, Zhang J, Yao P, Tai YL, Yuan Y. Cloning and functional analysis of three aphid alarm pheromone genes from German chamomile (*Matricaria chamomilla L.*). Plant Sci. 2020; 294: 110463.

[31] Andrew M, Mwafaq I, Shelby E, Mossab Y, Bhagwat N, Suzanne L, Douglas S, Philipp S, Susan RW, Dorothea T. Diversity and function of terpene synthases in the production of carrot aroma and flavor compounds. Sci Rep. 2020; 10: 9989.

[32] Kurin I, Jun-Ichiro H, Norihiko M, Kazutoshi S. cDNA cloning and functional analyses of ashitaba (*Angelica keiskei*) sesquiterpene synthase genes. J Oleo Sci. 2020; 69:711-718.

[33] Bhagwat N, Liora SH, Mosaab Y, Anas K, Rika K, Tejas CB, Mwafaq I. Characterization of terpene synthase genes potentially involved in black fig fly (*Silba adipata*) interactions with Ficus carica. Plant Sci. 2020; 298: 110549.

[34] Lian DM, Li WG, Yan XG, Qinggele C, Zhao GR, Qiao JJ. Molecular and functional evolution of the spermatophyte sesquiterpene synthases. Int J Mol Sci. 2021; 22: 6348.

[35] Chuang CW, Wen CH, Wu TJ, Li CC, Nien TC, Ma LT,Chen LH, Gene ST, Chin CT, Yi RL, Fang HC. Sesquiterpene synthases of *Zanthoxylum ailanthoides*: sources of unique aromas of a folklore plant in Taiwan. J Agric Food Chem. 2021; 69: 12494-12504.

[36] Sean A, Fernando LG, Claudia SD. Diversity of sesquiterpene synthases in the basidiomycete *Coprinus cinereus*. Molecular Microbiology. 2009; 72: 1181-1195.

[37] Siemon T, Wang ZQ, Bian GK, Seitz T, Ye ZL, Lu Y, Cheng S, Ding YK, Huang YL, Deng ZX, Liu TG, Christmann M. Semisynthesis of plant-derived englerin A enabled by microbe engineering of guaia-6,10(14)-diene as building block. J Am Chem Soc. 2020; 142:2760-2765.

[38] Zhang YP, Wang J, Wang ZB, Zhang YM, Shi SB, Nielsen J, Liu ZH. A gRNA-tRNA array for CRISPR-Cas9 based rapid multiplexed genome editing in *Saccharomyces cerevisiae.* Nat Commun. 2019; 5:1053.
